# Evaluation of Information Theoretic Network Meta-analysis to Rank First-Line Anticancer Regimens for Hormone Receptor–Positive, *ERBB2*-Negative Metastatic Breast Cancer

**DOI:** 10.1001/jamanetworkopen.2022.4361

**Published:** 2022-04-13

**Authors:** Xuanyi Li, Alicia Beeghly-Fadiel, Suresh K. Bhavnani, Hossein Tavana, Samuel M. Rubinstein, Bishal Gyawali, Irbaz Bin Riaz, H. Deepika Fernandes, Jeremy L. Warner

**Affiliations:** 1 Department of Medicine, Vanderbilt University Medical Center, Nashville, Tennessee; 2 Division of Epidemiology, Department of Medicine, Vanderbilt University Medical Center, Nashville, Tennessee; 3 Vanderbilt-Ingram Cancer Center, Nashville, Tennessee; 4 Department of Biomedical Informatics, University of Texas Medical Branch, Galveston; 5 Department of Biomedical Engineering, University of Akron, Akron, Ohio; 6 Lineberger Comprehensive Cancer Center, University of North Carolina, Chapel Hill; 7 Division of Cancer Care and Epidemiology, Departments of Oncology and Public Health Sciences, Queen’s University, Kingston, Ontario, Canada; 8 Department of Oncology, Dana Farber Cancer Institute, Boston, Massachusetts; 9 Division of Hematology and Oncology, Department of Medicine, Mayo Clinic, Phoenix, Arizona; 10 Edith Sanford Breast Center at Sanford Health, Bismarck, North Dakota; 11 Division of Hematology and Oncology, Department of Medicine, Vanderbilt University Medical Center, Nashville, Tennessee; 12 Department of Biomedical Informatics, Vanderbilt University, Nashville, Tennessee

## Abstract

**Importance:**

Hormone receptor–positive, *ERBB2 *(formerly *HER2*/neu)-negative metastatic breast cancer (HR-positive, *ERBB2*-negative MBC) is treated with targeted therapy, endocrine therapy, chemotherapy, or combinations of these modalities; however, evaluating the increasing number of treatment options is challenging because few regimens have been directly compared in randomized clinical trials (RCTs), and evidence has evolved over decades. Information theoretic network meta-analysis (IT-NMA) is a graph theory–based approach for regimen ranking that takes effect sizes and temporality of evidence into account.

**Objective:**

To examine the performance of an IT-NMA approach to rank HR-positive, ERBB2-negative MBC treatment regimens.

**Data Sources:**

HemOnc.org, a freely available medical online resource of interventions, regimens, and general information relevant to the fields of hematology and oncology, was used to identify relevant RCTs.

**Study Selection:**

All primary and subsequent reports of RCTs of first-line systemic treatments for HR-positive, ERBB2-negative MBC that were referenced on HemOnc.org and published between 1974 and 2019 were included. Additional RCTs that were evaluated by a prior traditional network meta-analysis on HR-positive, ERBB2-negative MBC were also included.

**Data Extraction and Synthesis:**

RCTs were independently extracted from HemOnc.org and a traditional NMA by separate observers. This study followed the Preferred Reporting Items for Systematic Reviews and Meta-analyses (PRISMA) reporting guideline for NMA with several exceptions: the risk of bias within individual studies and inconsistency in the treatment network were not assessed.

**Main Outcomes and Measures:**

Regimen rankings generated by IT-NMA based on clinical trial variables, including primary end point, enrollment number per trial arm, *P* value, effect size, years of enrollment, and year of publication.

**Results:**

A total of 203 RCTs with 63 629 patients encompassing 252 distinct regimens were compared by IT-NMA, which resulted in 151 rankings as of 2019. Combinations of targeted and endocrine therapy were highly ranked, especially the combination of endocrine therapy with cyclin-dependent kinase 4 and 6 (CDK4/6) inhibitors. For example, letrozole plus palbociclib was ranked first and letrozole plus ribociclib, third. Older monotherapies that continue to be used in RCTs in comparator groups, such as anastrozole (251 of 252) and letrozole (252), fell to the bottom of the rankings. Many regimens gravitated toward indeterminacy by 2019.

**Conclusions and Relevance:**

In this network meta-analysis study, combination therapies appeared to be associated with better outcomes than monotherapies in the treatment of HR-positive, ERBB2-negative MBC. These findings suggest that IT-NMA is a promising method for longitudinal ranking of anticancer regimens from RCTs with different end points, sparse interconnectivity, and decades-long timeframes.

## Key Points

**Question:**

Can information theoretic network meta-analysis (IT-NMA) rank the estimated efficacy of regimens for hormone receptor–positive, *ERBB2*-negative metastatic breast cancer (HR-positive, ERBB2-negative MBC)?

**Findings:**

In this network meta-analysis study, a combination of targeted and endocrine therapy, ie, letrozole and palbociclib, had the highest ranking. Rarely used regimens’ rank scores gravitated to indeterminacy, while monotherapies that compared unfavorably with novel agents or combinations in recent trials, such as anastrozole, had low rankings.

**Meaning:**

In this study, combination therapies were ranked more highly than monotherapies for treating HR-positive, ERBB2-negative MBC.

## Teaser

This study examines the performance of an information theoretic network meta-analysis approach to ranking treatment regimens for hormone receptor–positive, *ERBB2-*negative metastatic breast cancer.

## Introduction

Cancer treatment has become increasingly sophisticated. This increased complexity of cancer treatment has contributed to a 31% decrease in the overall cancer death rate from 1991 to 2018.^1^ However, keeping current with the increasing number of treatment options is challenging for health care practitioners, researchers, policy makers, and guideline developers.

Given the large number of cancer treatment regimens available, prospective head-to-head comparisons of all regimens are infeasible. Expert-driven clinical guidelines, such as those from the National Comprehensive Cancer Network (NCCN), often serve as the reference standard for regimen selection. But a potential limitation is that recommendations may be informed by opinions and clinical experiences of the guideline development group.^2^ Meta-analysis integrates findings from multiple trials and can considerably increase statistical power and resolve disparate findings among studies.^3^ Network meta-analysis (NMA) is a ranking technique that combines direct and indirect evidence within a network of clinical trials.^4^ Traditional NMA (tNMA) requires that the compared trials have the same clinical end point and does not account for time-varying trends, such as changes in prevalence, underlying biologic factors, obsolescence of certain treatment approaches, and evolving supportive care measures. We previously developed an approach to address some of the shortcomings of tNMA using information theoretic NMA (IT-NMA).^5,6^ Information theory is a broad array of techniques that provide quantitative measures of information content.^7^ We adapted concepts of information theory to model networks of randomized clinical trials (RCTs) as a distributed power grid where the “charge” of a regimen corresponds directly to its ranking, and the “impedance” of the “transmission line” between regimens corresponds to both the recency of the evidence and the surrogacy of the comparison. Regimens with comparatively superior efficacy gain charge at the expense of those with comparatively inferior efficacy, and these changes are propagated through the network; this propagation is made more difficult for older regimens to account for information decay over time. As an analogy to power distribution networks, older transmission lines accumulate impurities over time that decrease their conductivity and increase their resistance.^8^

Currently, breast cancer has the highest incidence and the second highest cancer mortality among US women.^9^ Hormone-receptor–positive, *ERBB2 *(formerly *HER2*/neu)-negative metastatic breast cancer (HR-positive, ERBB2-negative MBC) is the most common subtype of metastatic breast cancer and is considered to be incurable.^10^ HR-positive, ERBB2-negative MBC has been studied through carefully designed RCTs of cytotoxic chemotherapy, endocrine therapy, targeted therapy, and their combinations since the 1970s. Because of a very large number of treatment options, HR-positive, ERBB2-negative MBC is a good candidate for testing IT-NMA–based regimen ranking. We therefore used IT-NMA to conduct the largest analysis of first-line HR-positive/*ERBB2-*negative MBC trials to date, to our knowledge.

## Methods

### Data Sources

This study followed the Preferred Reporting Items for Systematic Reviews and Meta-analyses (PRISMA) reporting guideline for NMA with several exceptions: the risk of bias within individual studies and inconsistency in the treatment network were not assessed. We evaluated all RCTs of first-line systemic treatments for HR-positive, ERBB2-negative MBC that were referenced on HemOnc.org, an expert-curated website that undergoes continuous review for standard-of-care systemic anticancer therapy,^11^ or included in a prior tNMA.^12^ To include older RCTs that did not specify breast cancer subtypes, we included all RCTs that were not specifically for *ERBB2*-positive or triple-negative MBC. HemOnc.org aims to include all phase 3 RCTs through a standardized approach that has been previously described^13^; it also includes randomized phase 2 RCTs with regimens that are considered to be standard of care and/or have been the basis of regulatory approval by the US Food and Drug Administration (FDA). All breast cancer pages of HemOnc.org were hand searched; the related HemOnc ontology was also used to automatically screen out ineligible studies.^14^ Eligible reports were reviewed independently by 2 authors (X.L. and J.L.W.), and disagreements were adjudicated through discussion to consensus.

### Statistical Analysis

The IT-NMA algorithm has been previously described and is inspired by power grid network analysis^6,15^; briefly, it ranks regimens taking several elements of trials into account, including the *P* value and effect size for the least surrogate outcome with *P* ≤ .10, number of patients enrolled in each group, and an aging coefficient to account for the salience of a particular regimen. We used reported *P* values when available; for studies that reported only confidence intervals, we calculated *P* values following the method of Altman and Bland.^16^ For effect sizes, we used hazard ratios for time-based end points, such as overall survival (OS) and progression-free survival (PFS), and odds ratios (ORs) for fixed end points, such as response rates; if the hazard ratio was not available, as was the case for many older studies, we substituted the ratio of median survival times. The aging coefficient was an exponential decay coefficient with a half-life of 5.5 years, which was set to a value of 1 at the end of trial enrollment; subsequent publications and value propagation events refreshed the coefficient by 1 half-life (to a maximum value of 1). Examples of network value propagation are shown in eFigure 1 in the Supplement. The overall score for a given regimen at any given year was the summation of values from all trials of that regimen up to that date. This unitless score can be interpreted as follows: (1) regimens with positive scores are considered recommendable; (2) regimens with negative scores are considered not recommendable; and (3) scores near zero are considered to be indeterminate. Indeterminacy can be the result of the passage of time since a regimen has been evaluated (obsolescence), the failure of a regimen to demonstrate statistical superiority (a negative trial), and/or superior results for a regimen being counteracted by inferior results for the regimen. In addition to the overall rank score, the aging coefficient is reported for each regimen and is incorporated into network visualizations using the alpha (transparency) channel. For longitudinal regimen ranking, the algorithm generated a network and rank list for each year since the year of publication for the oldest RCTs in the network. The code to generate rankings is freely available on GitHub.^17^ The analysis was conducted in R version 4.1.1 (R Project for Statistical Computing) and also used R packages igraph version 1.2.8 and RColorBrewer version 1.1-2.

## Results

**Figure 1.**
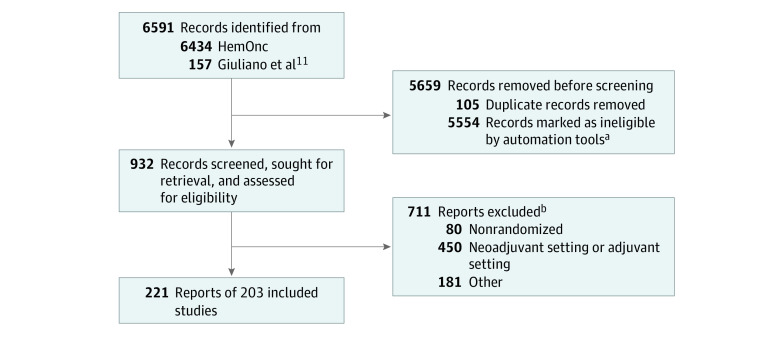
Study Flow Diagram of Randomized Clinical Trials in the Information Theoretic Network Meta-analysis for First-Line Treatment for Hormone Receptor–Positive, *ERBB2-*Negative Metastatic Breast Cancer ^a^Publications in domains other than breast cancer. ^b^Nonrandomized studies and studies in the adjuvant and neoadjuvant setting were determined using automatic tools. Other reasons for exclusion included studies of *ERBB2-*positive or triple-negative breast cancer as well as studies in non–first-line settings.

Beginning with a total of 6591 records (6434 from HemOnc.org and 157 from a prior tNMA), a total of 203 RCTs that enrolled 63 629 patients from 1972 onwards that were published between 1974 and 2019 were identified and included in this IT-NMA (Figure 1). The RCTs included 134 phase 3 trials, 27 randomized phase 2 trials, and 42 studies that did not specify phase; 172 were identified from HemOnc.org and 31 were from the prior tNMA.^12^ Overall, 24 trials (77%) not present on HemOnc.org were randomized phase 2 trials. Information about included trials, including reference, study type, population, regimens, years of enrollment and publication, and study end point are provided in eTable 1 in the Supplement. Considering variations in dose, frequency, and number of cycles, there were 252 unique regimens, including 149 cytotoxic chemotherapies, 37 endocrine therapies, 15 combination endocrine and targeted therapies, 31 combination cytotoxic chemotherapy and targeted therapies, 17 combination chemotherapy and endocrine therapies, and 3 other therapies, including observation.

**Figure 2.**
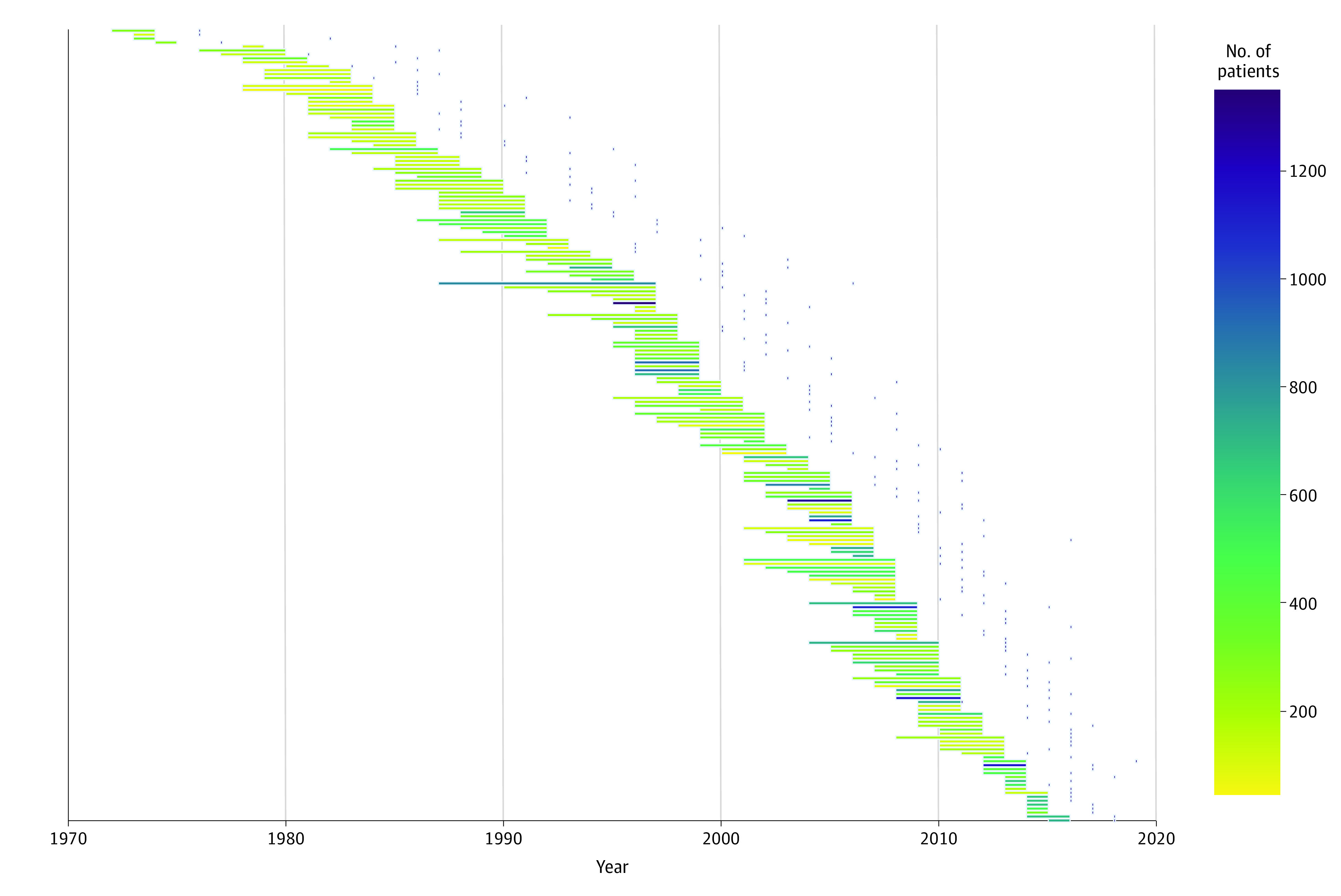
Gantt Plot of Included Randomized Clinical Trials Horizontal bars represent periods of enrollment. Colors of the bars represent the number of patients enrolled. Dots after the bars represent the year of the first publication from the RCT. Intervals between end of enrollment and first publication vary considerably.

The median (IQR) number of patients per RCT was 236 (148-414), and the median (IQR) period of enrollment was 3 (2-4) years (Figure 2). Intervals between the end of enrollment and first publication varied; although the median (IQR) was 4 (3-5) years, some trials did not publish their results until many years later.

**Figure 3.**
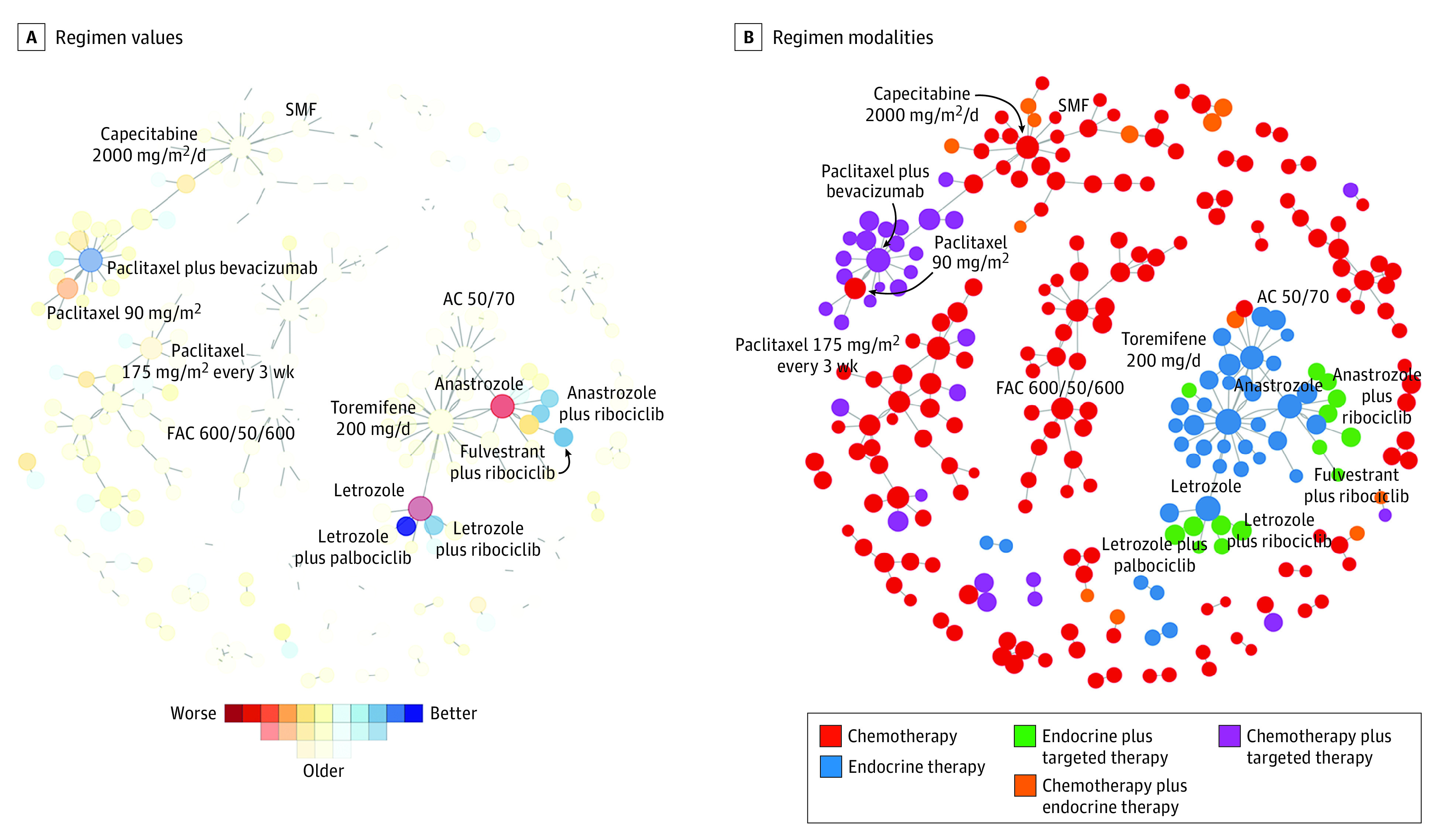
Information Theoretic Network Meta-analysis Regimen Network for Hormone Receptor–Positive, *ERBB2-*Negative Metastatic Breast Cancer, as of 2019 Nodes are regimens, and edges are randomized comparisons. (A) The color of a node reflects its rank score, and the color scale is shown beneath the figure. This highlights that most of the nodes in the network are faded, indicating a low aging coefficient. Nonfaded nodes cluster in 2 areas of the network, with the highly ranked endocrine therapy with cyclin-dependent kinase 4 and 6 inhibitor regimens between 3 and 6 o’clock, and paclitaxel plus bevacizumab in the cluster at 10 o’clock. It can also be appreciated that the network is not fully connected. (B) The color of a node reflects its modality. Cytotoxic chemotherapy regimens dominate the network, reflecting a diversity of treatment approaches that evolved throughout the 1970s and 1980s. Between 3 and 6 o’clock, a dense cluster of endocrine therapy regimens has many endocrine plus targeted therapy regimens at its edges, indicative of the tendency to compare new combination therapies with existing single-agent endocrine therapy regimens. At 10 o’clock, a cluster of cytotoxic chemotherapy plus targeted therapy regimens is seen to surround paclitaxel plus bevacizumab. It can be seen that the connection between the endocrine therapy–dominated cluster and the cytotoxic chemotherapy–dominated clusters is nonexistent. AC 50/750 indicates doxorubicin 50 mg/m^2^ plus cyclophosphamide 750 mg/m^2^; FAC 600/50/600, fluorouracil 600 mg/m^2^ plus doxorubicin 50 mg/m^2^ plus cyclophosphamide 600 mg/m^2^; SMF, prednimustine, methotrexate, fluorouracil.

Figure 3 shows the network of regimens for the year 2019. After accounting for ties, there were 151 regimen ranks in 2019. The Table includes the 54 regimens with an aging coefficient of 0.5 or greater; the full list of ranked regimens and their modalities is provided in eTable 2 in the Supplement. Four of the top 5 regimens were combinations of endocrine therapy and cyclin-dependent kinase 4 and 6 inhibitors (CDK4/6i). For example, letrozole plus palbociclib was ranked first and letrozole plus ribociclib, third. Most of the lowest-ranked regimens were monotherapies, such as letrozole (last of 252) and anastrozole (251); the lowest-ranked combination regimens with aging coefficients of 0.5 or greater were vinorelbine plus capecitabine and weekly ixabepilone plus bevacizumab. eFigure 2 in the Supplement illustrates the distribution of rank scores vs aging coefficients. Many regimens gravitated toward indeterminacy by 2019.

**Table.** Ranked Regimens With an Aging Coefficient of at Least 0.5

Rank	Regimen	Value	Aging coefficient	Modality
1	Letrozole plus palbociclib	17.84	0.91	Endocrine and targeted therapy
2	Paclitaxel plus bevacizumab	15.92	0.64	Cytotoxic chemotherapy and targeted therapy
3	Letrozole plus ribociclib	11.87	0.78	Endocrine and targeted therapy
4	Anastrozole plus ribociclib	10.88	0.88	Endocrine and targeted therapy
5	Fulvestrant plus ribociclib	9.31	1.00	Endocrine and targeted therapy
6	Abemaciclib plus anastrozole	9.30	0.78	Endocrine and targeted therapy
7	Capecitabine plus paclitaxel plus bevacizumab	6.13	0.69	Cytotoxic chemotherapy and targeted therapy
8	Capecitabine 2000 mg/m^2^ plus docetaxel 75 mg/m^2^, 8 cycles	4.77	0.60	Cytotoxic chemotherapy
12	Anastrozole plus fulvestrant	3.81	0.65	Endocrine therapy
13	Docetaxel 60 to 75 mg/m^2^, every 3-4 weeks	3.66	0.69	Cytotoxic chemotherapy
14	Letrozole plus bevacizumab	3.33	0.69	Endocrine therapy and targeted therapy
15	Paclitaxel 80 mg/m^2^, 3 weeks out of 4	3.20	1.00	Cytotoxic chemotherapy
16 (tie)	Docetaxel plus PLD	3.01	0.69	Cytotoxic chemotherapy
16 (tie)	Polymeric micellar paclitaxel	3.01	0.78	Cytotoxic chemotherapy
20	Capecitabine plus vinorelbine plus bevacizumab	2.24	0.69	Cytotoxic chemotherapy and targeted therapy
24	Docetaxel plus ramucirumab	1.87	0.78	Cytotoxic chemotherapy and targeted therapy
26	Sapitinib 20 mg 2/d plus anastrozole	1.55	0.69	Endocrine therapy and targeted therapy
29	Gemcitabine plus paclitaxel	1.34	0.51	Cytotoxic chemotherapy
32	Capecitabine 2000 mg/m^2^/d, limited duration	1.14	0.53	Cytotoxic chemotherapy
47 (tie)^a^	Paclitaxel plus bevacizumab plus gemcitabine	0.65	0.69	Cytotoxic chemotherapy and targeted therapy
57	Sapitinib 40 mg 2/d plus anastrozole	0.48	0.69	Endocrine therapy and targeted therapy
60	Taxane plus bevacizumab	0.41	0.60	Cytotoxic chemotherapy and targeted therapy
67	nab-Paclitaxel 150 mg/m^2^	0.28	0.63	Cytotoxic chemotherapy
82 (tie)^a^	Pictilisib plus paclitaxel	0.11	0.69	Cytotoxic chemotherapy and targeted therapy
95	Paclitaxel plus bevacizumab plus everolimus	0.05	0.69	Cytotoxic chemotherapy and targeted therapy
98 (tie)^a^	Trebananib 10 mg/kg plus paclitaxel plus bevacizumab	0.02	0.58	Cytotoxic chemotherapy and targeted therapy
110 (tie)	Buparlisib plus paclitaxel	0.00	0.78	Cytotoxic chemotherapy and targeted therapy
110 (tie)	Docetaxel 75 mg/m^2^ plus bevacizumab 15 mg/kg	0.00	0.57	Cytotoxic chemotherapy and targeted therapy
110 (tie)	Docetaxel plus bevacizumab, followed by exemestane plus bevacizumab	0.00	0.60	Cytotoxic chemotherapy, endocrine therapy, and targeted therapy
110 (tie)	Paclitaxel plus sunitinib	0.00	0.60	Cytotoxic chemotherapy and targeted therapy
110 (tie)	Sunitinib plus paclitaxel plus bevacizumab	0.00	0.69	Cytotoxic chemotherapy and targeted therapy
110 (tie)	Ixabepilone every 3 wk plus bevacizumab	0.00	0.69	Cytotoxic chemotherapy and targeted therapy
187 (tie)^a^	Trebananib 3 mg/kg plus paclitaxel plus bevacizumab	−0.19	0.56	Cytotoxic chemotherapy and targeted therapy
197 (tie)^a^	Capecitabine 2500 mg/m^2^/d	−0.30	0.69	Cytotoxic chemotherapy
202	Taxane plus bevacizumab plus capecitabine	−0.41	0.60	Cytotoxic chemotherapy and targeted therapy
210 (tie)^a^	Trebananib 10 mg/kg plus paclitaxel	−0.57	0.56	Cytotoxic chemotherapy and targeted therapy
223	Sepantronium plus docetaxel	−1.12	0.60	Cytotoxic chemotherapy and targeted therapy
224 (tie)^a^	PLD 45 mg/m^2^	−1.14	0.53	Cytotoxic chemotherapy
226	Docetaxel 75 mg/m^2^	−1.18	0.74	Cytotoxic chemotherapy
227	Capecitabine plus bevacizumab	−1.26	0.66	Cytotoxic chemotherapy and targeted therapy
228	Capecitabine plus bevacizumab plus cyclophosphamide	−1.27	0.69	Cytotoxic chemotherapy and targeted therapy
232	Docetaxel 30 mg/m^2^	−1.80	0.60	Cytotoxic chemotherapy
233	NPLD plus vinorelbine	−1.98	0.53	Cytotoxic chemotherapy
234	nab-Paclitaxel plus bevacizumab	−2.01	0.60	Cytotoxic chemotherapy and targeted therapy
242	Micellar paclitaxel	−3.20	1.00	Cytotoxic chemotherapy
243	S-1	−3.66	0.69	Cytotoxic chemotherapy
245	Fulvestrant 500 mg	−4.11	0.94	Endocrine therapy
246	Vinorelbine plus capecitabine	−4.77	0.64	Cytotoxic chemotherapy
247	Ixabepilone weekly plus bevacizumab	−6.24	0.59	Cytotoxic chemotherapy and targeted therapy
248	Capecitabine 2000 mg/m^2^/d	−7.03	0.57	Cytotoxic chemotherapy
250	Paclitaxel 90 mg/m^2^	−11.17	0.62	Cytotoxic chemotherapy
251	Anastrozole	−29.36	0.70	Endocrine therapy
252	Letrozole	−33.27	0.54	Endocrine therapy

Abbreviations: NPLD, non-pegylated liposomal doxorubicin; PLD, pegylated liposomal doxorubicin; S-1: tegafur, gimeracil, and oteracil.

^a^ The other tied regimens are not shown here given that their aging coefficients were less than 0.5; see eTable 2 in the Supplement, where all regimens are listed.

**Figure 4.**
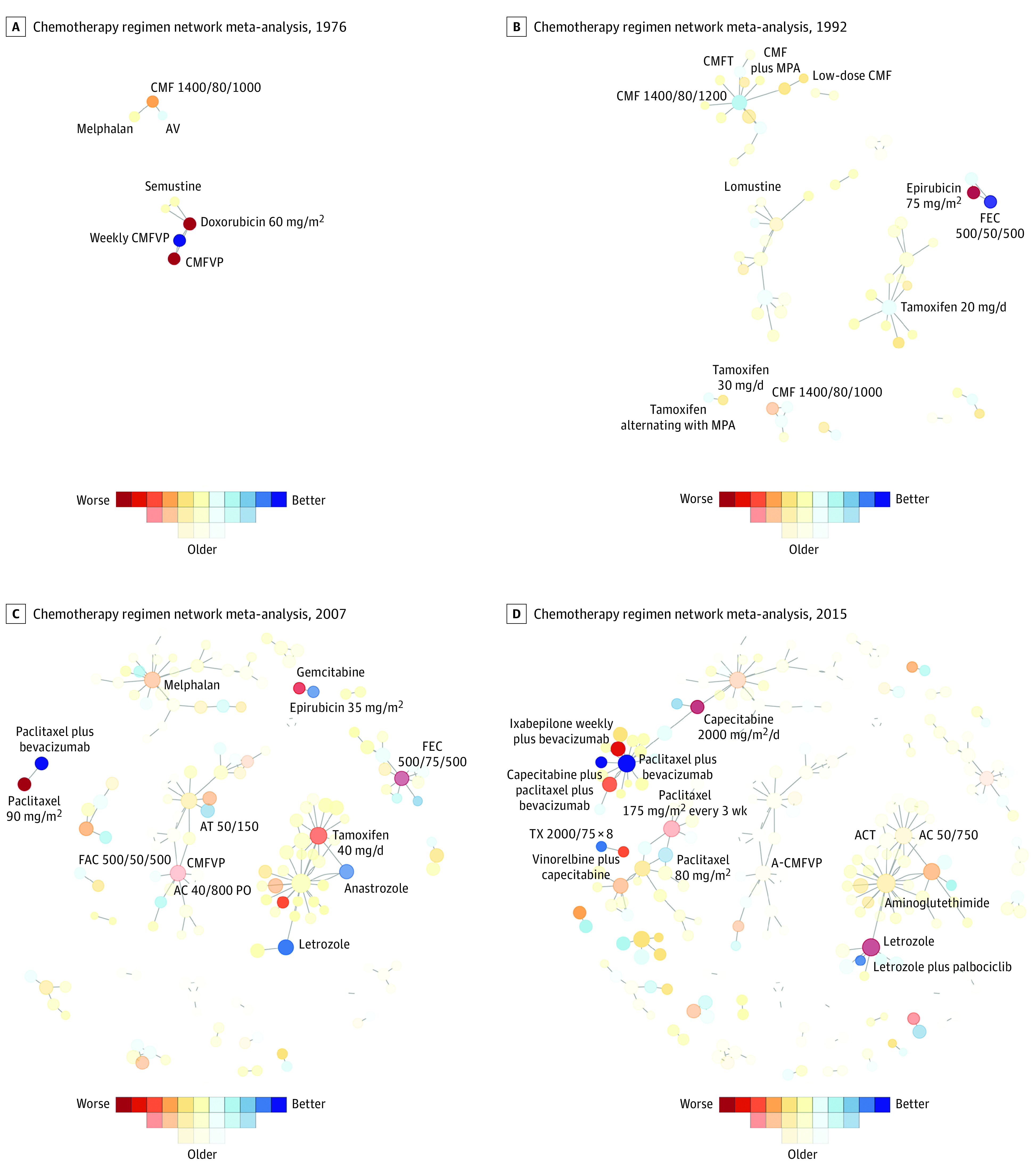
Snapshots of the Information Theoretic Network Meta-analysis Regimen Network for Hormone Receptor–Positive, *ERBB2-*Negative Metastatic Breast Cancer in 1976, 1992, 2007, and 2015 A, In 1976, the small network was dominated by chemotherapy regimens, with weekly CMFVP (cyclophosphamide, methotrexate, fluorouracil, vincristine, prednisone) ranked highest. B, In 1992, single-agent tamoxifen was positively ranked, as were the triple-drug regimens CMF (cyclophosphamide, methotrexate, fluorouracil) and FEC (fluorouracil, epirubicin, cyclophosphamide) in other, unconnected clusters. C, In 2007, Aromatase inhibitors (anastrozole and letrozole) replaced tamoxifen. Paclitaxel plus bevacizumab was also new to the network and highest ranked. D, In 2015, letrozole plus palbociclib debuted and rose toward the top of the rankings; separately, paclitaxel plus bevacizumab remained highly ranked. A-CMFVP indicates doxorubicin followed by CMFVP; AC 40/800 PO, doxorubicin 40 mg/m^2^ plus oral cyclophosphamide 800 mg/m^2^; AC 50/750, doxorubicin 50 mg/m^2^ plus cyclophosphamide 750 mg/m^2^; ACT, doxorubicin plus cyclophosphamide plus tamoxifen; AT 50/150, doxorubicin 50 mg/m^2^ plus paclitaxel 150 mg/m^2^; AV, doxorubicin plus vincristine; CMF 1400/80/1000, oral cyclophosphamide 1400 mg/m^2^ plus methotrexate 80 mg/m^2^ plus fluorouracil 1000 mg/m^2^; CMF 1400/80/1200, oral cyclophosphamide 1400 mg/m^2^ plus methotrexate 80 mg/m^2^ plus fluorouracil 1200 mg/m^2^; CMFT, cyclophosphamide, methotrexate, fluorouracil, tamoxifen; FAC 500/50/500, fluorouracil 500 mg/m^2^ plus doxorubicin 50 mg/m^2^ plus cyclophosphamide 500 mg/m^2^; FEC 500/75/500, fluorouracil 500 mg/m^2^ plus epirubicin 75 mg/m^2^ plus cyclophosphamide 500 mg/m^2^; MPA, medroxyprogesterone acetate; TX 2000/75 × 8, capecitabine 2000 mg/m^2^ plus docetaxel 75 mg/m^2^, 8 cycles.

Figure 4 shows snapshots of the regimen networks in the years of 1974, 1994, 2007, and 2018. Since the publication of the first RCTs on treatments for MBC in 1974,^18^ many RCTs and regimens have been added to the network, and clusters began to emerge. When a novel treatment regimen was tested in escalation trials (ie, higher dose intensity and/or additional drugs in the experimental group), it tended to rise to the top of the rank list. For example, the combination regimen of paclitaxel and bevacizumab came to near the top of the rankings in 2007, which coincides with the publication of the ECOG E2100 trial.^19^ This trial was the basis of accelerated approval of bevacizumab for metastatic breast cancer in 2008. We visualized the changing landscape of the regimen network from 1974 to 2019 (a span of 45 years) in videos, links to which are provided in the eAppendix in the Supplement.

## Discussion

We evaluated 252 unique treatment regimens for HR-positive, ERBB2-negative MBC from 203 RCTs by IT-NMA. This is by far the largest such comparison that we are aware of; the aforementioned largest published tNMA evaluated 149 regimens. Results from the current study of RCTs through 2019 showed that combinations of targeted and endocrine therapy ranked highest, and monotherapies, such as letrozole, ranked lowest.

Current NCCN guidelines recommend the combination of an aromatase inhibitor or selective ER down-regulator and a CDK4/6i as first-line therapy for HR-positive, ERBB2-negative MBC as category 1, meaning that based on high-level evidence, there is uniform NCCN consensus that the intervention is appropriate.^20^ Our results agree with this recommendation. Endocrine monotherapies, such as letrozole, are listed as first-line other recommended regimens by NCCN, although the recommendation is category 2A, meaning that it is based on lower-level evidence, but there is still uniform NCCN consensus that the intervention is appropriate.^20^ These regimens were at the bottom of our ranked list. A possible explanation is that because the older endocrine monotherapies are still recognized as a treatment option, clinical trials continue to use them in control groups. As an example, single-agent letrozole was used in the control group in 10 RCTs evaluated; all experimental groups in these trials were 2 drug combinations. In 9 of these RCTs, the control group was comparatively inferior. As previously reported, our algorithm can partially address but not completely overcome such “straw man” effects in regimen networks.^6^ Future work will explore the incorporation of scores that account for toxic effects, such as the ESMO Magnitude of Clinical Benefit Scale,^21^ which may offset the tendency to reward escalation trial designs, which usually trade efficacy for toxic effects.

Treatment for HR-positive, ERBB2-negative MBC has evolved. Since the late 1970s, endocrine therapy regimens, such as anastrozole, exemestane, fulvestrant, goserelin, letrozole, tamoxifen, and toremifene, have been investigated for the treatment of HR-positive breast cancers.^22,23,24,25,26,27,28^ In more recent years, targeted therapies such as palbociclib, ribociclib, and abemaciclib, which target the cyclin-D–CDK4/6–Rb pathway, have been tested. These have been shown to act synergistically with antiestrogens to inhibit the growth of HR-positive breast cancer.^29^ The combination of targeted and endocrine therapies has been shown to be efficacious and has led to improvements in both response rates and survival.^30,31,32,33,34^ On the other hand, chemotherapy has remained a mainstay for the treatment of MBC. But the addition of targeted therapies to cytotoxic chemotherapies has yielded equivocal results. The addition of bevacizumab to paclitaxel led to improvement in PFS but not OS in ECOG E2100, and bevacizumab’s FDA indication for breast cancer was subsequently withdrawn; however, the European Medicines Agency indication remains.^19,35^ Despite this, the regimen was highly ranked by our analysis because of positive PFS results in 2 subsequently published phase 3 RCTs: CALGB 40502 and MERiDiAN.^36,37^ Notably, similar findings to ours were reported in a traditional NMA by Giuliano et al.^12^ These authors theorized that the finding may be in part because of the inclusion of patients with triple-negative breast cancer in some trials^38^; this possibility cannot be disambiguated from methodologic issues in the absence of individual patient-level analysis. Despite the common use of surrogate end points in oncology RCTs, the bevacizumab finding is discrepant with prevailing clinical opinion. Although this highlights issues with a clinical trial literature that largely relies on surrogate end points, it also suggests that further methodological refinements are needed in the IT-NMA approach.

Conversely, IT-NMA enables ranking of chemotherapy and endocrine therapy regimens together, even though they are rarely directly compared (Figure 3B). The network distance between these distinct treatment approaches can be vast, suggesting that probabilistic methods, such as tNMA, may be less reliable or even impossible to execute if the network is not completely interconnected.

Many regimens have been discarded over time, with rank scores gravitating toward zero due to the aging coefficient in our IT-NMA algorithm.^6^ Primarily because of this obsolescence, more than half of the regimens in the rank list were indeterminate, with a rank score near zero. Given that these regimens have no contemporary comparisons, it cannot be concluded that they are any better or worse than currently studied regimens; in fact, some could even be candidates for repurposing in the future. However, there are some older monotherapy regimens that continue to be used in control groups in more recent clinical trials. Because these older regimens performed unfavorably in trials, they sank to the bottom of our rank list, meaning that they remained salient. For example, tamoxifen at 20 mg/d was ranked 220 of 252. This regimen was first tested in 1977,^23^ and the latest trial with this regimen in the control group was published in 2018—a span of more than 4 decades.^33^ Our finding provides evidence supporting the concern that suboptimal regimens in control groups may be used in many RCTs. The use of a substandard comparator regimen may result in a trial that is more likely to be positive but actually prevents the trial from addressing the clinically relevant question of whether a new drug is better than the current standard of care.^39^

Our regimen ranking results are in accordance with findings from Giuliano et al^12^; however, that study included both first-line and second-line studies, whereas the current study was restricted to the first-line setting only, potentially increasing the clinical relevance of our findings. Nevertheless, it must be acknowledged that substantial heterogeneity remains in the characteristics of the patients studied, specifically regarding prior treatment exposure. De novo MBC is rare,^40,41,42^ such that most patients with MBC had early-stage disease at diagnosis and either had a late recurrence or primary disease progression. In many cases, these patients will have received endocrine and/or cytotoxic chemotherapy in the adjuvant or neoadjuvant setting.

IT-NMA has several advantages when compared with other regimen ranking methods, such as those used in Giuliano et al.^12^ One of the challenges associated with meta-analysis of oncology clinical trials is heterogenous outcomes. Our IT-NMA approach can construct a network containing RCTs with different primary end points, using empirically derived coefficients for different primary end points, eg, 1 for OS, and 0.8 for PFS. Other regimen ranking methods, such as the American Society of Clinical Oncology Value Framework, use comparable coefficients, although future exploration of varying coefficients for surrogate end points is warranted in light of the bevacizumab findings noted previously.^43^ More importantly, IT-NMA allows longitudinal regimen ranking. For HR-positive, ERBB2-negative MBC, our IT-NMA produced a rank list of regimens for each year from 1974 to 2019, which enabled us to examine how the field has evolved over time. Given the successful ranking of regimens for HR-positive, ERBB2-negative MBC, we plan to use IT-NMA to rank regimens for other common and extensively studied cancers.

### Limitations

This study has limitations. One of the challenges of this study was visualizing the regimen network. To date, most published NMAs have used circular layouts; however, this layout can become cluttered and less informative with increasing numbers of nodes and edges. To illustrate this, we have reconstructed the circular network as reported by Giuliano et al^12^ using a force-directed layout (eFigure 3 in the Supplement). The regimen network in this reanalysis contained 273 nodes and 237 edges and was visualized with the Fruchterman-Rheingold force-based layout.^44^ As this layout shows, chemotherapy- and endocrine therapy–based regimens are clearly segregated, with just a tenuous connection between the 2 dominant components by way of 2 rarely used and relatively understudied regimens (mitoxantrone monotherapy and everolimus plus exemestane). We believe that, compared with a circular layout, a force-based layout can lead to a greater understanding of the complexity of a high-dimensional space of indirect comparisons. Next steps include building interactive approaches for the visualization of regimen networks.

Furthermore, because the chemotherapy and endocrine therapy subnetworks are essentially disconnected, direct comparisons of chemotherapy and endocrine therapy–based regimens should be undertaken with caution. We did not incorporate toxic effects into our IT-NMA algorithm, such that our rankings are primarily driven by efficacy. As mentioned previously, value-based frameworks such as the ESMO Magnitude of Clinical Benefit Scale^21^ can present a more balanced picture between efficacy and toxic effects and will be incorporated into future iterations. Although we attempted to be exhaustive in our search for RCTs, it is likely that some trials were missed, especially non–English language trials and older trials that may be less discoverable using current literature searching techniques. Additionally, we concede that the published literature is biased toward trials with positive results, exacerbating the tendency to overvalue experimental groups and undervalue control groups. Mandatory clinical trial registration may alleviate this tendency, although adherence to registration has become uniform only in the last decade. We also did not evaluate several issues with clinical trial design and implementation that could skew the outcomes, such as the appropriateness of the control regimen or crossover designs.^45^

## Conclusions

In this study, combination therapies were more highly ranked than endocrine therapy or chemotherapy alone for treating HR-positive, ERBB2-negative MBC, with combination CDK4/6i and endocrine therapy at the top of our ranked list. Our findings demonstrate that IT-NMA is a promising method for direct and indirect comparisons to rank cancer treatment regimens and their evolution over time. Although these results are best considered preliminary, we anticipate that with continued refinement the IT-NMA approach will eventually demonstrate clinical utility.

## Notes

Supplement. **eFigure 1.** Illustration of Value Propagation Through The Regimen Network **eFigure 2.** Plot of Regimen Rank Score vs Aging Coefficient **eFigure 3.** Reconstructed Network of Metastatic Breast Cancer Trials in Giuliano et al, 2019, Using a Force-Directed Layout **eTable 1.** Information on RCTs included in This IT-NMA **eTable 2.** Full Ranked List of Regimens by IT-NMA **eAppendix. **Videos

## References

[1] Siegel RL, Miller KD, Fuchs HE, Jemal A. Cancer statistics, 2021. CA Cancer J Clin. 2021;71(1):7-33. doi:10.3322/caac.21654 33433946

[2] Woolf SH, Grol R, Hutchinson A, Eccles M, Grimshaw J. Clinical guidelines: potential benefits, limitations, and harms of clinical guidelines. BMJ. 1999;318(7182):527-530. doi:10.1136/bmj.318.7182.527 10024268 PMC1114973

[3] Lee YH. An overview of meta-analysis for clinicians. Korean J Intern Med. 2018;33(2):277-283. doi:10.3904/kjim.2016.195 29277096 PMC5840596

[4] Rouse B, Chaimani A, Li T. Network meta-analysis: an introduction for clinicians. Intern Emerg Med. 2017;12(1):103-111. doi:10.1007/s11739-016-1583-7 27913917 PMC5247317

[5] Warner J, Yang P, Alterovitz G. Automated synthesis and visualization of a chemotherapy treatment regimen network. Stud Health Technol Inform. 2013;192:62-66. 23920516 PMC4075319

[6] Warner JL, Yang PC, Alterovitz G. Overcoming the straw man effect in oncology: visualization and ranking of chemotherapy regimens using an information theoretic approach. JCO Clin Cancer Inform. 2017;1(1):1-9. doi:10.1200/CCI.17.00079 30657401 PMC6874021

[7] Ghahramani Z. Information theory. In: Encyclopedia of Cognitive Science. American Cancer Society; 2006. doi:10.1002/0470018860.s00643

[8] Jeon S-J. Definitions of apparent power and power factor in a power system having transmission lines with unequal resistances. IEEE Trans Power Deliv. 2005;20(3):1806-1811. doi:10.1109/TPWRD.2005.848658

[9] Siegel RL, Miller KD, Jemal A. Cancer statistics, 2020. CA Cancer J Clin. 2020;70(1):7-30. doi:10.3322/caac.21590 31912902

[10] DeSantis CE, Ma J, Gaudet MM, . Breast cancer statistics, 2019. CA Cancer J Clin. 2019;69(6):438-451. doi:10.3322/caac.21583 31577379

[11] Warner JL, Cowan AJ, Hall AC, Yang PC. HemOnc.org: a collaborative online knowledge platform for oncology professionals. J Oncol Pract. 2015;11(3):e336-e350. doi:10.1200/JOP.2014.001511 25736385 PMC5706141

[12] Giuliano M, Schettini F, Rognoni C, . Endocrine treatment versus chemotherapy in postmenopausal women with hormone receptor-positive, *HER2*-negative, metastatic breast cancer: a systematic review and network meta-analysis. Lancet Oncol. 2019;20(10):1360-1369. doi:10.1016/S1470-2045(19)30420-6 31494037

[13] Li X, Sigworth EA, Wu AH, . Seven decades of chemotherapy clinical trials: a pan-cancer social network analysis. Sci Rep. 2020;10(1):17536. doi:10.1038/s41598-020-73466-6 33067482 PMC7568560

[14] Warner JL, Dymshyts D, Reich CG, . HemOnc: a new standard vocabulary for chemotherapy regimen representation in the OMOP common data model. J Biomed Inform. 2019;96:103239. doi:10.1016/j.jbi.2019.103239 31238109 PMC6697579

[15] Pagani GA, Aiello M. The power grid as a complex network: a survey. Phys Stat Mech Its Appl. 2013;392(11):2688-2700. doi:10.1016/j.physa.2013.01.023

[16] Altman DG, Bland JM. How to obtain the *P* value from a confidence interval. BMJ. 2011;343:d2304. doi:10.1136/bmj.d2304 22803193

[17] GitHub. IT-NMA. Accessed February 21, 2022. https://github.com/lix321994/IT-NMA.git

[18] Gottlieb JA, Rivkin SE, Spigel SC, . Proceedings: superiority of andomized over oral nitrosoureas in patients with advanced breast carcinoma. A Southwest Cancer Chemotherapy study Group study. Cancer. 1974;33(2):519-526. doi:10.1002/1097-0142(197402)33:2<519::AID-CNCR2820330229>3.0.CO;2-X 4812769

[19] Miller K, Wang M, Gralow J, . Paclitaxel plus bevacizumab versus paclitaxel alone for metastatic breast cancer. N Engl J Med. 2007;357(26):2666-2676. doi:10.1056/NEJMoa072113 18160686

[20] Development and Update of Guidelines. National Comprehensive Cancer Network. Accessed May 27, 2021. https://www.nccn.org/guidelines/guidelines-process/development-and-update-of-guidelines

[21] Cherny NI, Dafni U, Bogaerts J, . ESMO-Magnitude of Clinical Benefit Scale version 1.1. Ann Oncol. 2017;28(10):2340-2366. doi:10.1093/annonc/mdx310 28945867

[22] Pyrhönen S, Valavaara R, Modig H, . Comparison of toremifene and tamoxifen in post-menopausal patients with advanced breast cancer: a randomized double-blind, the ‘nordic’ phase III study. Br J Cancer. 1997;76(2):270-277. doi:10.1038/bjc.1997.375 9231932 PMC2223944

[23] Ingle JN, Ahmann DL, Green SJ, . Randomized clinical trial of diethylstilbestrol versus tamoxifen in postmenopausal women with advanced breast cancer. N Engl J Med. 1981;304(1):16-21. doi:10.1056/NEJM198101013040104 7001242

[24] Mouridsen H, Gershanovich M, Sun Y, . Superior efficacy of letrozole versus tamoxifen as first-line therapy for postmenopausal women with advanced breast cancer: results of a phase III study of the International Letrozole Breast Cancer Group. J Clin Oncol. 2001;19(10):2596-2606. doi:10.1200/JCO.2001.19.10.2596 11352951

[25] Jonat W, Kaufmann M, Blamey RW, . A andomized study to compare the effect of the andomized hormone releasing hormone (LHRH) analogue goserelin with or without tamoxifen in pre- and perimenopausal patients with advanced breast cancer. Eur J Cancer. 1995;31A(2):137-142. doi:10.1016/0959-8049(94)00415-2 7718316

[26] Howell A, Robertson JFR, Abram P, . Comparison of fulvestrant versus tamoxifen for the treatment of advanced breast cancer in postmenopausal women previously untreated with endocrine therapy: a multinational, double-blind, randomized trial. J Clin Oncol. 2004;22(9):1605-1613. doi:10.1200/JCO.2004.02.112 15117982

[27] Paridaens RJ, Dirix LY, Beex LV, . Phase III study comparing exemestane with tamoxifen as first-line hormonal treatment of metastatic breast cancer in postmenopausal women: the European Organisation for Research and Treatment of Cancer Breast Cancer Cooperative Group. J Clin Oncol. 2008;26(30):4883-4890. doi:10.1200/JCO.2007.14.4659 18794551 PMC2652082

[28] Bonneterre J, Thürlimann B, Robertson JF, . Anastrozole versus tamoxifen as first-line therapy for advanced breast cancer in 668 postmenopausal women: results of the Tamoxifen or Arimidex Randomized Group Efficacy and Tolerability study. J Clin Oncol. 2000;18(22):3748-3757. doi:10.1200/JCO.2000.18.22.3748 11078487

[29] Finn RS, Dering J, Conklin D, . PD 0332991, a selective cyclin D kinase 4/6 inhibitor, preferentially inhibits proliferation of luminal estrogen receptor-positive human breast cancer cell lines in vitro. Breast Cancer Res. 2009;11(5):R77. doi:10.1186/bcr2419 19874578 PMC2790859

[30] Finn RS, Martin M, Rugo HS, . Palbociclib and letrozole in advanced breast cancer. N Engl J Med. 2016;375(20):1925-1936. doi:10.1056/NEJMoa1607303 27959613

[31] Hortobagyi GN, Stemmer SM, Burris HA, . Ribociclib as first-line therapy for HR-positive, advanced breast cancer. N Engl J Med. 2016;375(18):1738-1748. doi:10.1056/NEJMoa1609709 27717303

[32] Slamon DJ, Neven P, Chia S, . Phase III randomized study of ribociclib and fulvestrant in hormone receptor-positive, human epidermal growth factor receptor 2-negative advanced breast cancer: MONALEESA-3. J Clin Oncol. 2018;36(24):2465-2472. doi:10.1200/JCO.2018.78.9909 29860922

[33] Tripathy D, Im SA, Colleoni M, . Ribociclib plus endocrine therapy for premenopausal women with hormone-receptor-positive, advanced breast cancer (MONALEESA-7): a andomized phase 3 trial. Lancet Oncol. 2018;19(7):904-915. doi:10.1016/S1470-2045(18)30292-4 29804902

[34] Goetz MP, Toi M, Campone M, . MONARCH 3: abemaciclib as initial therapy for advanced breast cancer. J Clin Oncol. 2017;35(32):3638-3646. doi:10.1200/JCO.2017.75.6155 28968163

[35] Sasich LD, Sukkari SR. The US FDAs withdrawal of the breast cancer indication for Avastin (bevacizumab). Saudi Pharm J. 2012;20(4):381-385. doi:10.1016/j.jsps.2011.12.001 23960813 PMC3744967

[36] Rugo HS, Barry WT, Moreno-Aspitia A, . Randomized phase III trial of paclitaxel once per week compared with nanoparticle albumin-bound nab-paclitaxel once per week or ixabepilone with bevacizumab as first-line chemotherapy for locally recurrent or metastatic breast cancer: CALGB 40502/NCCTG N063H (Alliance). J Clin Oncol. 2015;33(21):2361-2369. doi:10.1200/JCO.2014.59.5298 26056183 PMC4500830

[37] Miles D, Cameron D, Bondarenko I, . Bevacizumab plus paclitaxel versus placebo plus paclitaxel as first-line therapy for HER2-negative metastatic breast cancer (MERiDiAN): a double-blind placebo-controlled andomized phase III trial with prospective biomarker evaluation. Eur J Cancer. 2017;70:146-155. doi:10.1016/j.ejca.2016.09.024 27817944

[38] Brodowicz T, Lang I, Kahan Z, . Selecting first-line bevacizumab-containing therapy for advanced breast cancer: TURANDOT risk factor analyses. Br J Cancer. 2014;111(11):2051-2057. doi:10.1038/bjc.2014.504 25268370 PMC4260030

[39] Hilal T, Gonzalez-Velez M, Prasad V. Limitations in clinical trials leading to anticancer drug approvals by the US Food and Drug Administration. JAMA Intern Med. 2020;180(8):1108-1115. doi:10.1001/jamainternmed.2020.2250 32539071 PMC7296449

[40] Cortesi L, Toss A, Cirilli C, . Twenty-years experience with de novo metastatic breast cancer. Int J Cancer. 2015;137(6):1417-1426. doi:10.1002/ijc.29503 25736070

[41] Mariotto AB, Etzioni R, Hurlbert M, Penberthy L, Mayer M. Estimation of the number of women living with metastatic breast cancer in the United States. Cancer Epidemiol Biomarkers Prev. 2017;26(6):809-815. doi:10.1158/1055-9965.EPI-16-0889 28522448 PMC5833304

[42] McKenzie HS, Maishman T, Simmonds P, Durcan L, Eccles D, Copson E; POSH Steering Group. Survival and disease characteristics of de novo versus recurrent metastatic breast cancer in a cohort of young patients. Br J Cancer. 2020;122(11):1618-1629. doi:10.1038/s41416-020-0784-z 32231292 PMC7250836

[43] Schnipper LE, Davidson NE, Wollins DS, . Updating the American Society of Clinical Oncology Value Framework: revisions and reflections in response to comments received. J Clin Oncol. 2016;34(24):2925-2934. doi:10.1200/JCO.2016.68.2518 27247218

[44] Fruchterman TMJ, Reingold EM. Graph drawing by force-directed placement. Softw Pract Exper. 1991;21(11):1129-1164. doi:10.1002/spe.4380211102

[45] Gyawali B, de Vries EGE, Dafni U, . Biases in study design, implementation, and data analysis that distort the appraisal of clinical benefit and ESMO-Magnitude of Clinical Benefit Scale (ESMO-MCBS) scoring. ESMO Open. 2021;6(3):100117. doi:10.1016/j.esmoop.2021.100117 33887690 PMC8086024

