# Supplementary material for: Evaluation of Information Theoretic Network Meta-analysis to Rank First-Line Anticancer Regimens for Hormone Receptor–Positive, ERBB2-Negative Metastatic Breast Cancer
Source: JAMA Netw Open. 2022 Apr 13;5(4):e224361. doi: 10.1001/jamanetworkopen.2022.4361 (PMC9008500; doi:10.1001/jamanetworkopen.2022.4361)
Supplement: Supplement. — eFigure 1. Illustration of Value Propagation Through The Regimen Network eFigure 2. Plot of Regimen Rank Score vs Aging Coefficient eFigure 3. Reconstructed Network of Metastatic Breast Cancer Trials in Giuliano et al, 2019, Using a Force-Directed Layout eTable 1. Information on RCTs included in This IT-NMA eTable 2. Full Ranked List of Regimens by IT-NMA eAppendix. Videos [file jamanetwopen-e224361-s001.pdf]

Supplemental Online Content

Li X, Beeghly-Fadiel A, Bhavnani SK, et al. Evaluation of information theoretic network meta-analysis to rank first-line anticancer regimens for hormone receptor–positive, *ERBB2*-negative metastatic breast cancer. *JAMA Netw Open.* 2022;5(4):e224361. doi:10.1001/jamanetworkopen.2022.4361

eFigure 1. Illustration of Value Propagation Through The Regimen Network

eFigure 2. Plot of Regimen Rank Score vs Aging Coefficient

eFigure 3. Reconstructed Network of Metastatic Breast Cancer Trials in Giuliano et al, 2019, Using a Force-Directed Layout

eTable 1. Information on RCTs included in This IT-NMA

eTable 2. Full Ranked List of Regimens by IT-NMA

eAppendix. Videos

This supplemental material has been provided by the authors to give readers additional information about their work.

© 2022 Li X et al. *JAMA Network Open.*

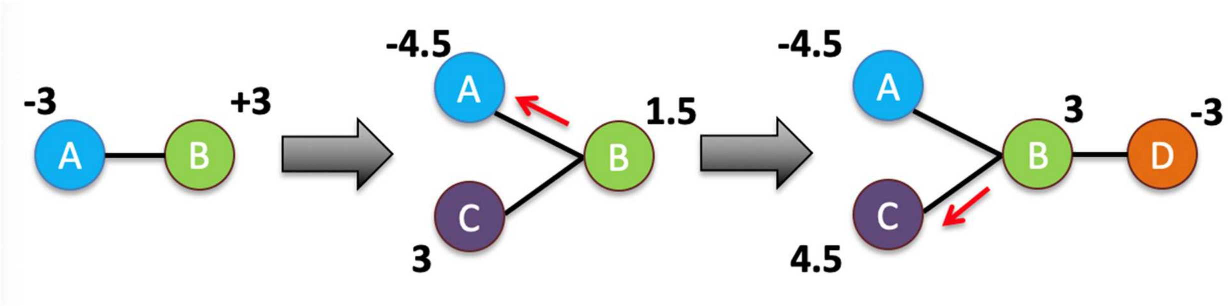

eFigure 1. Illustration of Value Propagation Through The Regimen Network

The network starts with one randomized controlled trial (RCT) comparing regimens A and B. When an additional RCT comparing regimen B and C is added to the network, B is inferior to C and its loss is transferred to A, which has previously been shown to be inferior to B. Then another trial comparing regimens B and D is added. Because B is superior to D, it will pass its gain back to C, which was previously shown to be superior to B.

© 2022 Li X et al. *JAMA Network Open.*

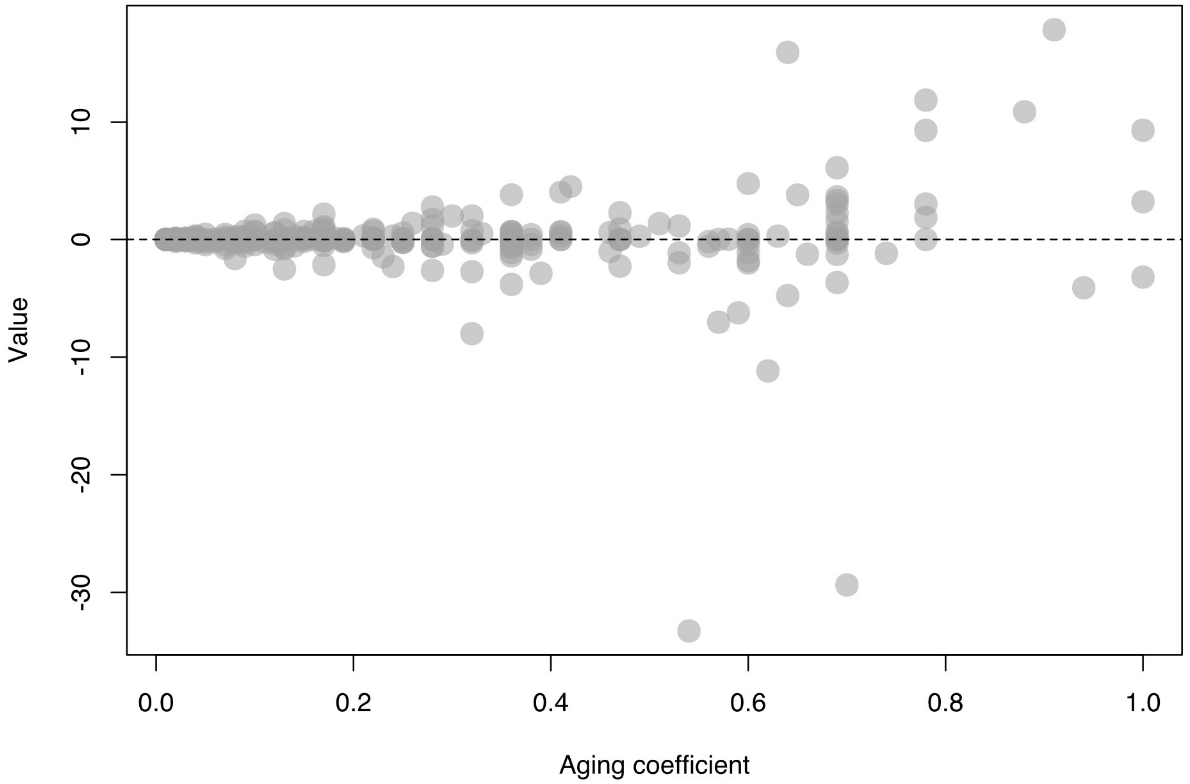

eFigure 2. Plot of Regimen Rank Score vs Aging Coefficient

Because the IT-NMA algorithm includes an aging coefficient, older regimens that have not been compared in more recent clinical trials tend to have scores closer to zero (with indeterminant rankings).

© 2022 Li X et al. *JAMA Network Open.*

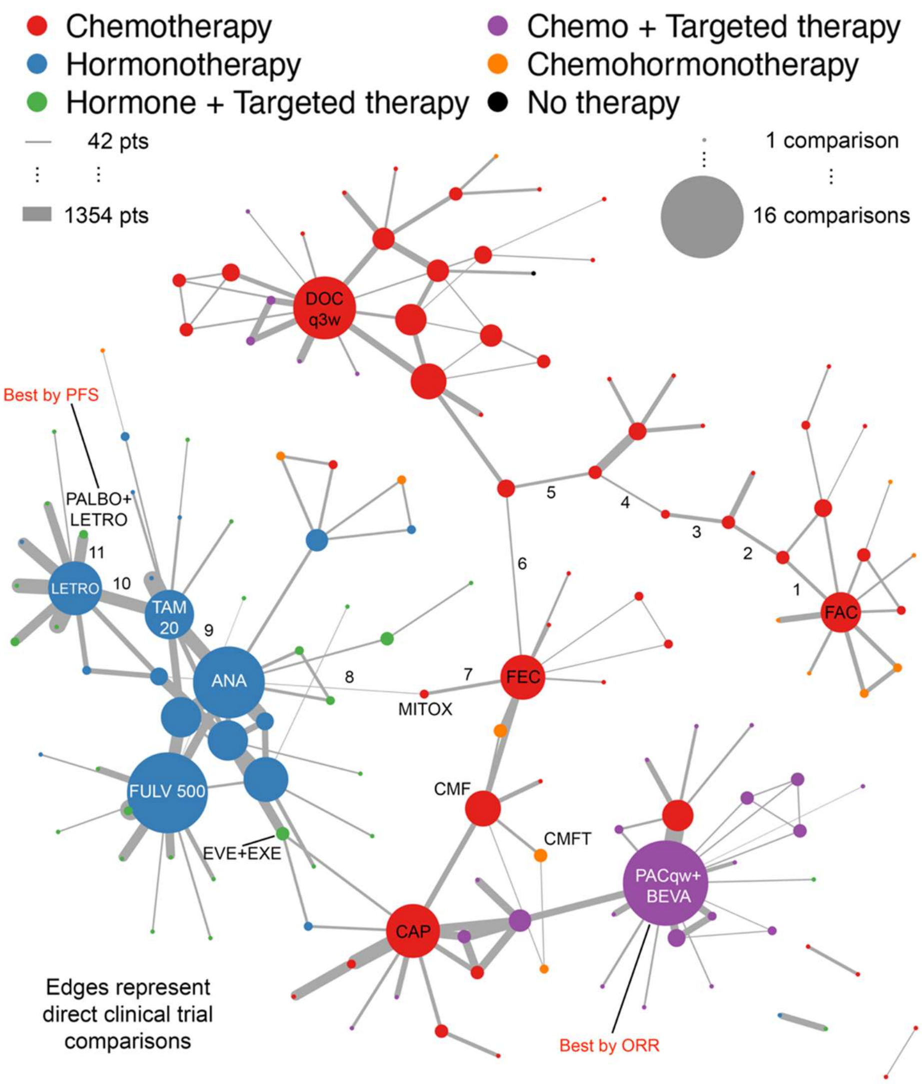

eFigure 3. Reconstructed Network of Metastatic Breast Cancer Trials in Giuliano et al, 2019, Using a Force-Directed Layout

Line width is proportional to the number of patients in pairwise comparisons; circle size is proportional to the number of direct comparisons to other regimens (degree). Common regimens, the two regimens connecting the chemotherapy and hormonotherapy components, the two regimens ascertained as best by progression-free survival (PFS) and by overall response rate (ORR) by the tNMA, and CMFT are labeled. Numbers represent the minimum path from FAC, a common chemotherapy regimen, and palbociclib & letrozole, the best regimen by PFS as ascertained by the tNMA. Abbreviations: ANA: anastrozole; BEVA: bevacizumab; CAP: capecitabine; CMF: cyclophosphamide, methotrexate, fluorouracil; CMFT: cyclophosphamide, methotrexate, fluorouracil, tamoxifen; DOC: docetaxel; EVE: everolimus; EXE: exemestane; FAC: fluorouracil, doxorubicin, cyclophosphamide; FEC: fluorouracil, epirubicin, cyclophosphamide; FULV 500: fulvestrant 500 mg; LETRO: letrozole; MITOX: mitoxantrone; qw: every week; q3w: every third week; PAC: paclitaxel; PALBO: palbociclib; TAM 20: tamoxifen 20 mg/day

© 2022 Li X et al. *JAMA Network Open.*

eTable 1. Information on RCTs included in This IT-NMA

Part 1: Initial publication 1974-1988

PMID	Study	Phase	Regimen 1 (R1)	R2	R3	Total N	R1 pts	R2 pts	R3 pts	Primary Pub Yr	1st Update	Enrollment Years	Endpoint
4812769	SWG02	3	Doxorubicin 60	Lomustine	Semustine	91	40	20	31	1974	-	1972	OS[1]
991103	Canellos et al. 1976	3	CMF 1400/80/1200	Melphalan	-	184	93	91	-	1976	-	NR	OS[1]
1260337	Brambilla et al. 1976	NR	AV	CMF 1400/80/1200	-	105	52	53	-	1976	-	1973-1974	OS
947510	Hoogstraten et al. 1976	NR	CMFVP weekly	CMFVP	Doxorubicin 60 q3wk	283	106	98	79	1976	-	1972-1974	ORR
6688538	Smalley et al. 1977	3	FAC 500/50/500	CMFVP	-	265	135	130	-	1977	1983	1974-1975	OS
348293	Bull et al. 1978	3	FAC 1000/60/1400 indefinite	CMF 1400/80/1200	-	78	38	40	-	1978	-	NR	OS
657081	Tranum et al. 1978	NR	AFCM	AF	FAC 800/40/400	313	105	105	103	1978	-	NR	OS[1]
387212	Hortobagyi et al. 1979	3	FAC-BCG	FAC 1000/50/500	-	149	105	44	-	1979	-	1974-NR	ORR
7001242	Ingle et al. 1981	NR	DES	Tamoxifen 20	-	143	74	69	-	1981	-	1977-1980	OS
7049347	ECOG E2173	NR	CMFP	AV	CMF 1400/80/1200	331	86	166	79	1982	-	1973-1974	OS[1]
7037152	SWOG 7405 B	NR	FAC 500/50/500	A-CMFVP	AC 40/800 PO	448	160	148	140	1982	-	NR	OS[1]
3655856	CALGB 8081	NR	CAFT	FAC 1000/50/1400	-	130	68	62	-	1983	1987	1980-1982	OS
6336981	Cocconi et al. 1983	NR	CMF 100/40/600	CMFT	-	145	74	71	-	1983	-	NR	OS
6380554	Bratherton et al. 1984	NR	Tamoxifen 20	Tamoxifen 40	-	237	116	121	-	1984	-	NR	ORR
6145832	Powles et al. 1984	NR	TAD	Tamoxifen 20	-	222	111	111	-	1984	-	1979-1983	ORR
3894587	Cummings et al. 1985	NR	FAC 1000/60/1400 maximum Doxorubicin500	CMFP	-	106	53	53	-	1985	-	1978-1979	OS[1]
3902200	Viladiu et al. 1985	NR	CMFT	CMF 1400/80/1200	CMF & MPA	117	40	38	39	1985	-	1978-1981	OS
3595668	Gundersen et al. 1986	3	VAC	Doxorubicin 20	-	128	66	62	-	1986	-	1982-1983	ORR
3528402	Buchanan et al. 1986	NR	Bilateral oophorectomy	Tamoxifen 40	-	111	53	58	-	1986	-	1979-1983	OS
2868074	Forbes et al. 1986	NR	ACT	Tamoxifen 40	AC 50/750	339	113	113	113	1986	-	1978-1981	OS[1]

© 2022 Li X et al. *JAMA Network Open.*

3519885	Ingle et al. 1986	NR	AG & HC & Tamoxifen	Tamoxifen 20	-	100	51	49	-	1986	-	1978-1984	OS
3511184	Ingle et al. 1986a	NR	Tamoxifen 20	Bilateral oophorectomy	-	53	26	27	-	1986	-	1978-1984	OS
2939943	van Veelen et al. 1986	NR	Tamoxifen 40	MPA	-	129	68	61	-	1986	-	1980-1984	OS[1]
3655855	Aisner et al. 1987	3	CAFVP	FAC 1000/50/1400	CMF 1400/80/1000	283	86	91	106	1987	-	1976-1980	ORR
3546611	Conte et al. 1987	NR	DES-CEF	FEC 600/60/600	-	116	57	59	-	1987	-	1983-1985	OS
7751892	ECOG E2177	NR	O+CAF	FAC 1000/60/1400 maximum Doxorubicin500	-	80	42	38	-	1987	1995	NR-1983	OS
3664426	Kellokumpu-Lehtinen et al. 1987	NR	Tamoxifen 40	Nandrolone	-	127	67	60	-	1987	-	1979-1983	OS[1]
3815266	Rosner et al. 1987	NR	CMFVP	CFP	AC 40/400	141	48	46	47	1987	-	1981-1985	OS[1]
2897433	Ambrosini et al. 1988	3	FAC 500/50/500	FEC 1000/50/500	-	443	221	222	-	1988	-	1983-1985	ORR
3049953	Bennett et al. 1988	3	FAC 500/50/500	CNF	-	331	164	167	-	1988	-	1983-1985	OS
3292710	Muss et al. 1988	3	Tamoxifen 20	Megestrol	-	125	64	61	-	1988	-	1981-1984	OS
3054453	SWOG S8020	3	Doxorubicin & Etoposide	Doxorubicin 60	-	115	56	59	-	1988	-	NR	OS
2458438	Tannock et al. 1988	3	CMF 600/40/600	Low-dose CMF	-	106	53	53	-	1988	-	1981-1986	ORR
1991261	Ingle et al. 1988	NR	Tamoxifen 20	Fluoxymesterone & Tamoxifen	-	238	119	119	-	1988	1991	1981-1985	OS[1]
3219274	Rubens et al. 1988	NR	Tamoxifen 20 & ovarian irradiation & Prednisolone	Tamoxifen 20 & ovarian irradiation	-	201	101	100	-	1988	-	1981-1986	OS[1]

[1]Only median is reported

Part 2: Initial publication 1990-2005

PMID	Study	Phase	Regimen 1 (R1)	R2	R3	R4	Total N	R1 pts	R2 pts	R3 pts	R4 pts	Primary Pub Yr	1st Update	2nd Update	Enrollment Years	Endpoint
2138477	Gundersen et al. 1990	3	Doxorubicin 20	Epirubicin 50	-	-	149	81	68	-	-	1990	-	-	1984-1986	ORR
2265137	Nielsen et al. 1990	3	Epirubicin 60	Epirubicin & Vindesine	-	-	143	76	67	-	-	1990	-	-	1983-1986	ORR
2078484	Cocconi et al. 1990	NR	CMF 1400/80/1200	CMF x 6 -> intensification	-	-	95	49	46	-	-	1990	-	-	1981-1985	OS
2151369	Gundersen et al. 1990a	NR	Tamoxifen/MPA	Tamoxifen 30	-	-	151	78	73	-	-	1990	-	-	NR	ORR
1988577	Bonneterre & Hurteloup 1991	3	FEC 500/75/500	FEC 500/50/500	Epirubicin 75	-	391	130	129	132	-	1991	-	-	NR	ORR
1832904	EORTC 10808	3	CMF 1400/80/1200	CMF 600/40/600	-	-	254	125	129	-	-	1991	-	-	1981-1984	OS

© 2022 Li X et al. *JAMA Network Open.*

1960557	Perez et al. 1991	3	Epirubicin 90	Doxorubicin 60	-	-	138	70	68	-	-	1991	-	-	1985-1988	ORR
2066763	Cocconi et al. 1991	NR	PE	CMF 1400/80/1200	-	-	130	65	65	-	-	1991	-	-	1985-1988	OS
2049750	Ingle et al. 1991	NR	Tamoxifen 20	Tamoxifen & Prednisolone	-	-	256	126	130	-	-	1991	-	-	1985-1989	OS
8445422	Blomqvist et al. 1993	3	FEC 500/60/500	FEC weekly	-	-	170	86	84	-	-	1993	-	-	1987-1991	OS[1]
8435205	Ejlertsen et al. 1993	3	FEC x 18mo	FEC 600/60/600	-	-	318	157	161	-	-	1993	-	-	1986-1989	OS
8315422	Focan et al. 1993	3	FEC 500/50/500	FEC 500/100/500	-	-	141	70	71	-	-	1993	-	-	1985-1990	ORR
8280653	Castiglione-Gertsch et al. 1993	NR	MPA	Tamoxifen 20	-	-	119	55	64	-	-	1993	-	-	1982-1985	TTP
8280654	Gill et al. 1993	NR	Megestrol	Tamoxifen 40	Megestrol & Tamoxifen	-	180	60	58	62	-	1993	-	-	1984-1989	OS
8518020	Yosef et al. 1993	NR	SMF	CMF 1400/80/1200	-	-	153	83	70	-	-	1993	-	-	1983-1987	OS
7946592	Tominaga et al. 1994	3	CAF & MPA	FAC 1000/60/1400 indefinite	-	-	199	103	96	-	-	1994	-	-	NR	OS[1]
8293400	Gale et al. 1994	NR	AG	Tamoxifen 20	-	-	216	108	108	-	-	1994	-	-	1977-NR	OS[1]
7880604	Gundersen et al. 1994	NR	Doxorubicin & MPA	Doxorubicin 20	-	-	218	107	111	-	-	1994	-	-	1987-1990	ORR
8040675	Muss et al. 1994	NR	MPA 1000	Tamoxifen 20	-	-	182	91	91	-	-	1994	-	-	1985-1990	OS
8168039	NCCTG 87-32-52	NR	CMF 600/40/600	DES-CEF	-	-	163	80	83	-	-	1994	-	-	1987-1991	OS
7751891	CALGB 8281	3	VATH/CMFVP	VATH	FAC 1000/40/500	-	497	162	168	167	-	1995	-	-	1982-1987	OS[1]
7595707	Hayes et al. 1995	NR	Toremifene 60	Tamoxifen 20	Toremifene 200	-	648	221	215	212	-	1995	-	-	1988-1991	ORR
7718316	Jonat et al. 1995	NR	Goserelin & Tamoxifen	Goserelin	-	-	318	159	159	-	-	1995	-	-	1988-1991	OS
8648369	Bastholt et al. 1996	3	Epirubicin 90	Epirubicin 135	Epirubicin 40	Epirubicin 60	263	64	58	75	66	1996	-	-	1987-1991	ORR
8839903	Conte et al. 1996	3	DES-CEF	FEC 600/60/600	-	-	258	130	128	-	-	1996	-	-	1985-1990	OS[1]
8648371	Dogliotti et al. 1996	3	Epirubicin & Lonidamine	Epirubicin 120	-	-	207	103	104	-	-	1996	-	-	1991-1993	ORR
8839901	SAKK 20/88	3	Tamoxifen 20	Fadrozole	-	-	212	107	105	-	-	1996	-	-	1988-1994	ORR
8943670	Stuart et al. 1996	3	Tamoxifen 20	Megestrol	-	-	139	67	72	-	-	1996	-	-	1985-1988	OS
9386869	Gershanovich et al. 1997	3	Toremifene 240	Tamoxifen 40	Toremifene 60	-	463	157	149	157	-	1997	-	-	1987-1992	ORR
9093724	HEPI 010	3	FEC 500/100/500	FEC 500/50/500	-	-	453	212	241	-	-	1997	-	-	1989-1992	OS[1]
9231932	Pyrhönen et al. 1997	3	Tamoxifen 40	Toremifene 60	-	-	415	201	214	-	-	1997	-	-	1986-1992	ORR
10561297	Bishop et al. 1999	3	Paclitaxel 200	CMFP	-	-	209	107	102	-	-	1999	-	-	1993-NR	OS
10091793	Blajman et al. 1999	3	Doxorubicin & Vinorelbine	FAC 500/50/500	-	-	170	85	85	-	-	1999	-	-	1991-1994	ORR

© 2022 Li X et al. *JAMA Network Open.*

10674879	Esteban et al. 1999	3	FEC 1000/60/1400	CNF	-	-	145	73	72	-	-	1999	-	-	1987-1993	ORR
10550134	NSABP B-26	3	Paclitaxel 250 q3wk	Paclitaxel 250 over 3 hours	-	-	563	284	279	-	-	1999	-	-	1994-1996	ORR
10637238	ECOG E3186	3	CAFTH	FAC 1000/60/1400 x 6	-	-	213	106	107	-	-	2000	-	-	1988-1992	TTF
10673513	EORTC 10923	3	Doxorubicin 75	Paclitaxel 200	-	-	331	165	166	-	-	2000	2000	-	1993-1996	PFS
10856098	NCIC CTG MA.8	3	Doxorubicin 60	Doxorubicin & Vinorelbine	-	-	300	149	151	-	-	2000	-	-	1992-1995	OS[1]
11138459	Nielsen et al. 2000	3	Cisplatin & Epirubicin	Epirubicin 70	-	-	155	74	81	-	-	2000	-	-	1987-1990	OS[1]
10885599	Pacini et al. 2000	3	EM	LEM	LFEC	FEC 500/75/500	305	80	74	73	78	2000	-	-	1991-1996	ORR
10760307	Stadtmauer et al. 2000	3	CMF 1400/80/1200	CMF x 4-6 -> HDT	-	-	184	83	101	-	-	2000	-	-	1990-1997	OS
11078488	Arimidex Study Group 2000	NR	Anastrozole	Tamoxifen 20	-	-	353	171	182	-	-	2000	-	-	1996-1998	TTP
11078487	TARGET	NR	Tamoxifen 20	Anastrozole	-	-	668	328	340	-	-	2000	-	-	1995-1998	TTP
11697835	O'Shaughnessy et al. 2001	2	Capecitabine 2510	CMF 600/40/600	-	-	95	62	33	-	-	2001	-	-	1996-1997	ORR
11230490	Batist et al. 2001	3	MC 60/600	AC 60/600	-	-	297	142	155	-	-	2001	-	-	1994-1998	ORR
11304774	Del Mastro et al. 2001	3	HD-CEF14	FEC 600/60/600	-	-	151	77	74	-	-	2001	-	-	1994-1997	ORR
11181656	HEPI 013	3	FEC 1000/100/800	CMF 1400/80/1200	-	-	460	223	237	-	-	2001	-	-	1990-1992	TTP
11352951	ILBCG	3	Letrozole	Tamoxifen 20	-	-	916	458	458	-	-	2001	2003	2007	1996-1999	TTP
11251000	Jassem et al. 2001	3	AT(Taxol) 50/220	FAC 500/50/500	-	-	267	134	133	-	-	2001	-	-	1996-1998	OS
11261827	Milla-Santos et al. 2001	3	Toremifene 60	Tamoxifen 40	-	-	217	106	111	-	-	2001	-	-	1996-1999	OS
11900215	Bajetta et al. 2002	3	Tamoxifen 20	Octreotide LAR & Tamoxifen	-	-	199	100	99	-	-	2002	-	-	1996-1998	PFS
12377958	Berruti et al. 2002	3	Cisplatin & Epirubicin	Epirubicin 120	Epirubicin & Lonidamine	-	278	92	93	93	-	2002	-	-	1995-1999	TTP
12088118	Buzdar et al. 2002	3	Tamoxifen 20	Droloxifene	-	-	1354	673	681	-	-	2002	-	-	1995-1997	TTP
12118025	EORTC 10961	3	AC 60/600	AT(Taxol) 60/200	-	-	275	137	138	-	-	2002	-	-	1996-1999	PFS
11815957	Harris et al. 2002	3	Doxorubicin 75	NPLD	-	-	224	116	108	-	-	2002	-	-	1992-1998	OS
12419743	Heidemann et al. 2002	3	FEC 500/50/500	Mitoxantrone	-	-	260	127	133	-	-	2002	-	-	1992-1997	OS[1]
12562650	Arpino et al. 2003	2	Idoxifene	Tamoxifen 20	-	-	219	108	111	-	-	2003	-	-	1996-1999	ORR
12721259	CALGB 9140	3	FAC 1000/40/500	FAC & Leucovorin	-	-	241	120	121	-	-	2003	-	-	1991-1995	ORR
12797397	Capotorto et al. 2003	3	TI FEC	FEC 500/75/500	TI MMM	-	135	44	45	46	-	2003	-	-	1995-1998	ORR
12586793	ECOG E1193	3	AT (Taxol) 50/150	Doxorubicin 60	Paclitaxel 175 CI	-	731	244	245	242	-	2003	-	-	1993-1995	ORR

© 2022 Li X et al. *JAMA Network Open.*

12796608	Milla-Santos et al. 2003	3	Anastrozole	Tamoxifen 40	-	-	238	121	117	-	-	2003	-	-		1997-1999	OS
12637459	TAX 306	3	AT(Taxotere) 50/75 x8	AC 60/600	-	-	429	214	215	-	-	2003	-	-		NR	TTP
15381937	Bonneterre et al. 2004	2	DE x 8	FEC 500/75/500	-	-	142	70	72	-	-	2004	-	-		1998-2000	ORR
15169793	CALGB 9342	3	Paclitaxel 210	Paclitaxel 175 q3wk	Paclitaxel 250 q3wk	-	469	156	158	155	-	2004	-	-		1994-NR	ORR
15367414	Chan et al. 2004	3	MC 75/600	EC 75/600	-	-	160	80	80	-	-	2004	-	-		1996-1997	TTP
15305399	Conte et al. 2004	3	E-P	EP 90/200	-	-	198	92	106	-	-	2004	-	-		1996-2001	ORR
15367413	Fountzilas et al. 2004	3	Carboplatin & Paclitaxel	EP 80/175	-	-	327	164	163	-	-	2004	-	-		1999-2002	OS
15226326	GEICAM-9903	3	A-D	AT(Taxotere) 50/75 x 6	-	-	144	75	69	-	-	2004	-	-		1999-2001	OS[1]
14998846	O'Brien et al. 2004	3	Doxorubicin 60	PLD 50	-	-	509	255	254	-	-	2004	-	-		1998-2000	PFS
15197192	SBG 9403	3	Epirubicin & Vinorelbine	Epirubicin 90	-	-	387	193	194	-	-	2004	-	-		1995-1999	PFS
15117982	Howell et al. 2004	NR	Tamoxifen 20	Fulvestrant 250	-	-	587	274	313	-	-	2004	-	-		1998-2000	TTP
16192591	Bontenbal et al. 2005	3	AT(Taxotere) 50/75 x6	FAC 500/50/500	-	-	216	109	107	-	-	2005	-	-		1997-2002	OS
16172456	CA012-0	3	nab-Paclitaxel 260	Paclitaxel 175 q3wk	-	-	454	229	225	-	-	2005	-	-		2001-2002	TTP
15735116	CECOG BM1	3	Gemcitabine & Epirubicin & Paclitaxel	FEC 500/90/500	-	-	259	124	135	-	-	2005	-	-		1999-2002	TTP
15821120	Feher et al. 2005	3	Epirubicin 35	Gemcitabine	-	-	382	191	191	-	-	2005	-	-		1996-1999	OS[1]
15659490	Schmid et al. 2005	3	AT(Taxol) 60/200	CME	-	-	93	45	48	-	-	2005	-	-		1998-2002	CRR
16293863	UKNCRI AB01	3	EC 75/600	EP 75/200	-	-	705	352	353	-	-	2005	-	-		1996-1999	PFS
16096436	von Minckwitz et al. 2005	3	BMF	CMF 1400/80/1200	-	-	345	162	183	-	-	2005	-	-		1996-2001	TTP[1]

[1]Only median is reported

Part 3: Initial publication 2006-2019

PMID	Study	Phase	Regimen 1 (R1)	R2	R3	R4	Total N	R1 pts	R2 pts	R3 pts	R4 pts	Primary Pub Yr	1st Update	2nd Update	3rd Update	Enrollment Years	Endpoint
17045796	EORTC 10863	3	Intermittent Tamoxifen & MPA	Intermittent Tamoxifen	Tamoxifen 40	-	828	276	276	276	-	2006	-	-	-	1987-1997	PFS
16622454	Pacilio et al. 2006	3	Docetaxel 100 x 6	DE indefinite	-	-	51	25	26	-	-	2006	-	-	-	2000-2003	OS
17971594	Biomed 777-CLP-29	3	Atamestane & Toremifene	Letrozole	-	-	865	434	431	-	-	2007	-	-	-	2002-2005	TTP
17971595	Deshmane et al. 2007	3	Tamoxifen 20	Arzoxifene	-	-	352	175	177	-	-	2007	-	-	-	NR	PFS
18160686	ECOG E2100	3	Paclitaxel & Bevacizumab	Paclitaxel 90	-	-	673	347	326	-	-	2007	-	-	-	2001-2004	PFS

© 2022 Li X et al. *JAMA Network Open.*

17611792	ERASME 3	3	AT(Taxol) 50/175	AT(Taxotere) 50/75 x 4	-	-	210	103	107	-	-	2007	-	-	-	2000-2004	OS[1]
18037940	Pegase 03	3	FEC -> HDC+SCT	FEC 500/100/500	-	-	179	88	91	-	-	2007	-	-	-	1995-2001	OS
18711184	Albain et al. 2008	3	Gemcitabine & Paclitaxel	Paclitaxel 175 q3wk	-	-	529	266	263	-	-	2008	-	-	-	1999-2002	OS
7749156	Alonso et al. 1995	3	CNF	FAC 600/50/600	-	-	110	55	55	-	-	2008	-	-	-	1988-1991	OS[1]
18375893	CALGB 9840	3	Paclitaxel 80 weekly	Paclitaxel 175 q3wk	-	-	735	350	385	-	-	2008	-	-	-	1998-NR	OS
19020973	CELAROM	3	Celecoxib & Exemestane	Exemestane	-	-	157	74	83	-	-	2008	-	-	-	2003-2004	PFS
18955454	EGF30001	3	Paclitaxel & Lapatinib	Paclitaxel 175 q3wk	-	-	579	291	288	-	-	2008	-	-	-	2004-2005	TTP
18794551	EORTC 10951	3	Exemestane	Tamoxifen 20	-	-	371	182	189	-	-	2008	-	-	-	1996-2002	PFS
18483853	Fountzilas et al. 2008	3	Carboplatin & Paclitaxel	Docetaxel & Gemcitabine	Paclitaxel 80 qwk	-	413	136	144	133	-	2008	-	-	-	2002-2006	OS
18300256	Rivera et al. 2008	3	Docetaxel 100	Docetaxel 40	-	-	125	62	63	-	-	2008	-	-	-	2001-2004	ORR
18025439	NCIC CTG MA.16	NR	FEC -> HDC+SCT	FEC 600/70/600	-	-	224	112	112	-	-	2008	-	-	-	1997-2000	OS
26371134	FIRST	2	Fulvestrant 500	Anastrozole	-	-	205	102	103	-	-	2009	2012	2015	-	NR	OS
19470941	Gradishar et al. 2009	2	nab-Paclitaxel 300	Docetaxel 100	nab-Paclitaxel 100	nab-Paclitaxel 150	300	76	74	76	74	2009	-	-	-	2005-2006	ORR
19933081	Yardley et al. 2009	2	Docetaxel 36	PLD 40	-	-	102	52	50	-	-	2009	-	-	-	2001-2007	ORR
19819914	B9E-MC-S241	3	Docetaxel & Gemcitabine	Docetaxel 100 indefinite	-	-	237	122	115	-	-	2009	-	-	-	2002-2006	TTF
19687336	DOXIL-BCA-3001	3	Docetaxel & PLD	Docetaxel 75	-	-	751	378	373	-	-	2009	-	-	-	2004-2006	TTP
20361253	GEICAM 2001-01	3	A-D-PLD	A-D	-	-	155	78	77	-	-	2009	-	-	-	NR	TTP
19906761	HORG CT/02.09	3	TX (Taxotere) 1900/75	DE indefinite	-	-	272	136	136	-	-	2009	-	-	-	2002-2007	TTP[1]
19254942	JCOG9802	3	AC/D	AC 40/500	Docetaxel 60 q3wk	-	433	147	139	147	-	2009	-	-	-	1999-2003	TTF
19786658	EGF30008	NR	Lapatinib & Letrozole	Letrozole	-	-	1286	642	644	-	-	2009	-	-	-	2003-2006	PFS
20215537	Cristofanilli et al. 2010	2	Anastrozole & Gefitinib	Anastrozole	-	-	93	43	50	-	-	2010	-	-	-	2004-2006	PFS
20497961	SABRE-B	2	Paclitaxel & Bevacizumab	Sunitinib & Paclitaxel & Bevacizumab	-	-	46	23	23	-	-	2010	-	-	-	2007-2008	PFS
20498403	AVADO	3	Docetaxel & Bevacizumab 100/15 x 9	Docetaxel & Bevacizumab 100/7.5	Docetaxel 100 x 9	-	736	247	248	241	-	2010	-	-	-	2006-2007	PFS
20089562	Blohmer et al. 2010	3	DE x 8	EC 90/600	-	-	236	125	111	-	-	2010	-	-	-	2000-2003	ORR
21358208	D4	3	AT(Taxotere) 50/75 x 8	AT(Taxotere) 50/75 qwk x 8	-	-	85	43	42	-	-	2010	-	-	-	2001-2008	OS[1]
21723792	Brufsky et al. 2011	2	Paclitaxel & Bevacizumab	Paclitaxel & Bevacizumab	-	-	187	93	94	-	-	2011	-	-	-	2006-2008	ORR

© 2022 Li X et al. *JAMA Network Open.*

			& Gemcitabine														
21136213	Ghosn et al. 2011	2	Docetaxel 25	Vinorelbine & Capecitabine 25/1650	-	-	70	29	41	-	-	2011	-	-	-	2004-2008	ORR
21220480	Osborne et al. 2011	2	Gefitinib & Tamoxifen	Tamoxifen 20	-	-	206	105	101	-	-	2011	-	-	-	2003-2006	PFS
21555686	Rugo et al. 2013	2	Docetaxel 80	Axitinib & Docetaxel	-	-	167	55	112	-	-	2011	-	-	-	NR	TTP
21429799	TRIO 010	2	Motesanib & Paclitaxel	Paclitaxel 90	Paclitaxel & Bevacizumab	-	282	91	94	97	-	2011	-	-	-	2006-2008	ORR
22094540	Vici et al. 2011	2	Docetaxel & Gemcitabine	TX(Taxotere) 2500/75	-	-	72	36	36	-	-	2011	-	-	-	2003-2006	ORR
22025143	ANZ 0001	3	Capecitabine 1300	CMF 1400/80/1200	-	-	325	216	109	-	-	2011	-	-	-	2001-2005	PFS
21084429	B9E-MC-S273	3	TX (Taxotere) 2000/75 indefinite	Docetaxel & Gemcitabine	-	-	475	236	239	-	-	2011	-	-	-	2002-2008	TTP
21358207	D2	3	Docetaxel 75	Docetaxel 30	-	-	481	295	186	-	-	2011	-	-	-	2001-2008	ORR
21734418	ERASME-4	3	TX (Taxotere) 2000/75 indefinite	DE indefinite	-	-	68	33	35	-	-	2011	-	-	-	2004-2007	ORR
22094937	Hatschek et al. 2011	3	TEX	EP 75/175	-	-	287	144	143	-	-	2011	-	-	-	2002-2007	PFS
22084374	Nielsen et al. 2011	3	Docetaxel & Gemcitabine	Docetaxel 100	-	-	337	170	167	-	-	2011	-	-	-	2001-2005	TTP
21383283	RIBBON-1	3	Capecitabine 2000	Taxane & Anthracycline	Capecitabine & Bevacizumab	Tax & Anthra & Bevacizumab	1237	206	207	409	415	2011	-	-	-	2005-2007	PFS
21569994	SUN 1094	3	Paclitaxel & Bevacizumab	Paclitaxel & Sunitinib	-	-	485	243	242	-	-	2011	-	-	-	2006-2009	PFS
22412143	Baselga et al. 2012	2	Capecitabine & Sorafenib	Capecitabine 2000	-	-	229	115	114	-	-	2012	-	-	-	2007-2008	PFS
22418699	Carlson et al. 2012	2	Anastrozole & Gefitinib	Fulvestrant & Gefitinib	-	-	141	72	69	-	-	2012	-	-	-	2003-2007	ORR
22370325	FACT	3	Anastrozole & Fulvestrant	Anastrozole	-	-	514	258	256	-	-	2012	-	-	-	2004-2008	TTP
23233719	HORIZON	3	Letrozole & Temsirolimus	Letrozole	-	-	1112	556	556	-	-	2012	-	-	-	2004-2006	PFS
22331954	SUN 1064	3	Docetaxel & Sunitinib	Docetaxel 100	-	-	593	296	297	-	-	2012	-	-	-	2007-2009	PFS
22853014	SWOG S0226	NR	Anastrozole & Fulvestrant	Anastrozole	-	-	694	349	345	-	-	2012	2019	-	-	2004-2009	OS
23649189	CA163-115	2	Ixabepilone q3wk & Bevacizumab	Paclitaxel & Bevacizumab	Ixabepilone qwk & Bevacizumab	-	123	45	32	46	-	2013	-	-	-	NR	ORR
22954665	Gradishar et al. 2013	2	Paclitaxel & Sorafenib	Paclitaxel 90	-	-	237	119	118	-	-	2013	-	-	-	2007-2009	PFS
Abstract only[2]	Paul et al. 2013	2	Dasatinib & Letrozole	Letrozole	-	-	120	57	63	-	-	2013	-	-	-	NR in abstract	CBR
23715630	A5991048	3	Exemestane	Anastrozole	-	-	298	149	149	-	-	2013	-	-	-	2005-2010	TTP

© 2022 Li X et al. *JAMA Network Open.*

23537313	B9E-IT-S376	3	Docetaxel & Gemcitabine	Gemcitabine & Paclitaxel	-	-	241	118	123	-	-	2013	-	-	-	2005-2010	ORR
23569309	KCSG-BR07-02	3	Gemcitabine & Paclitaxel	Observation	-	-	231	116	115	-	-	2013	-	-	-	2007-2010	OS
23771714	Luck et al. 2013	3	Capecitabine & Paclitaxel	EP 60/175	-	-	340	170	170	-	-	2013	-	-	-	2002-2005	PFS
27501767	TURANDOT	3	Paclitaxel & Bevacizumab	Capecitabine & Bevacizumab	-	-	531	266	265	-	-	2013	2016	-	-	2008-2010	OS
25747197	Dieras et al. 2014	2	Trebananib 10 & Paclitaxel & Bevacizumab	Paclitaxel & Bevacizumab	Trebananib 3 & Paclitaxel & Bevacizuma b	Trebanani b 10 & Paclitaxel	228	56	58	57	57	2014	-	-	-	NR	PFS
25459393	Lam et al. 2014	2	Capecitabine & Paclitaxel & Bevacizumab	Paclitaxel & Bevacizumab	-	-	312	156	156	-	-	2014	-	-	-	2007-2010	PFS
25524798	PALOMA-1/TRIO-18	2	Letrozole & Palbociclib	Letrozole	-	-	165	84	81	-	-	2014	-	-	-	2009-2012	PFS
25176223	Lorusso et al. 2014	3	MC 60/600	NPLD & Vinorelbine	-	-	233	117	116	-	-	2014	-	-	-	2006-2011	TTP
24504445	OMEGA	3	Capecitabine 2000 limited duration	PLD 45	-	-	78	38	40	-	-	2014	-	-	-	2007-2011	PFS
25547219	Clemens et al. 2015	2	Docetaxel 75	Sepantronium & Docetaxel	-	-	101	51	50	-	-	2015	-	-	-	NR	PFS
26456573	Yardley et al. 2015	2	Paclitaxel & Bevacizumab & Everolimus	Paclitaxel & Bevacizumab	-	-	113	56	57	-	-	2015	-	-	-	2009-2011	PFS
26056183	CALGB 40502/NCCT G N063H	3	Paclitaxel & Bevacizumab	nab-Paclitaxel & Bevacizumab	Ixabepilone qwk & Bevacizuma b	-	783	267	275	241	-	2015	-	-	-	2008-2011	PFS
25691671	LEA	3	Letrozole & Ribociclib	Letrozole	-	-	374	190	184	-	-	2015	-	-	-	2007-2011	PFS
26096296	ML25241	3	TX(Taxotere) 2000/75 x8	Vinorelbine & Capecitabine	-	-	206	104	102	-	-	2015	-	-	-	2010-2013	PFS
25185099	ROSE/TRIO-12	3	Docetaxel & Ramucirumab	Docetaxel 75 q3wk	-	-	1144	759	385	-	-	2015	-	-	-	2008-2011	PFS
26617202	SELECT BC	3	Docetaxel 60-75 q3-4wk	S-1	-	-	618	309	309	-	-	2015	2016	-	-	2006-2010	OS
25519041	TABEA	3	Taxane & Bevacizumab	TBX	-	-	227	116	111	-	-	2015	-	-	-	2009-2012	PFS
27654971	MINT	2	Sapitinib 40 & Anastrozole	Sapitinib 20 & Anastrozole	Anastrozole	-	359	120	118	121	-	2016	-	-	-	2010-2012	PFS
27756583	NorCap-CA223	2	Gemcitabine & Paclitaxel	Vinorelbine & Capecitabine	Docetaxel & Gemcitabine	-	149	50	49	50	-	2016	-	-	-	2007-2009	DCR
27573562	PEGGY	2	Pictilisib & Paclitaxel	Paclitaxel 90	-	-	183	91	92	-	-	2016	-	-	-	2013-2014	PFS
27138575	CALGB 40503	3	Letrozole & Bevacizumab	Letrozole	-	-	343	173	170	-	-	2016	-	-	-	2008-2011	PFS
26927446	CARIN	3	Capecitabine & Vinorelbine & Bevacizumab	Capecitabine & Bevacizumab	-	-	592	295	297	-	-	2016	-	-	-	2009-2012	PFS
27908454	FALCON	3	Fulvestrant 500	Anastrozole	-	-	462	230	232	-	-	2016	-	-	-	2012-2014	PFS

© 2022 Li X et al. *JAMA Network Open.*

26916095	GINECO-BR107	3	Docetaxel & Bevacizumab 75/15 indefinite	Docetaxel & Bevacizumab -> Exemestane & Bevacizumab	-	-	117	59	58	-	-	2016	-	-	-	2010-2013	PFS6
27618821	GPMBC301	3	Genexol-PM	Paclitaxel 175 q3wk	-	-	212	105	107	-	-	2016	-	-	-	2008-2013	ORR
29174181	MERiDiAN	3	Paclitaxel & Bevacizumab	Paclitaxel 90	-	-	481	242	239	-	-	2016	2017	-	-	2012-2013	PFS
27717303	MONALEESA-2	3	Letrozole & Ribociclib	Letrozole	-	-	668	334	334	-	-	2016	2017	2018	2018	2014-2015	PFS
30632023	PALOMA-2	3	Letrozole & Palbociclib	Letrozole	-	-	666	444	222	-	-	2016	2019	-	-	2013-2014	PFS
27798749	PELICAN	3	PLD 50	Capecitabine 2500	-	-	210	105	105	-	-	2016	-	-	-	2006-2010	TTP
27724870	SAKK 24/09	3	Paclitaxel & Bevacizumab	Capecitabine & Bevacizumab & Cyclophosphamide	-	-	147	73	74	-	-	2016	-	-	-	2010-2012	OS
27803006	BELLE-4	3	Buparlisib & Paclitaxel	Paclitaxel 80 qwk	-	-	416	207	209	-	-	2017	-	-	-	2012-2014	PFS
28968163	MONARCH 3	3	Abemaciclib & Anastrozole	Anastrozole	-	-	493	328	165	-	-	2017	-	-	-	2014-2015	PFS
28256066	Tamura et al. 2017	3	Docetaxel 75	nab-Paclitaxel 150	-	-	197	99	98	-	-	2017	-	-	-	2009-2012	PFS
29860922	MONALEESA-3	3	Fulvestrant & Ribociclib	Fulvestrant 500	-	-	726	484	242	-	-	2018	2019	-	-	2015-2016	OS
29804902	MONALEESA-7	3	Anastrozole & Ribociclib	Anastrozole	-	-	672	335	337	-	-	2018	-	-	-	2014-2016	PFS
30745582	A3105301	3	Paclitaxel 80; 3 weeks out of 4	NK105 (micellar paclitaxel)	-	-	422	211	211	-	-	2019	-	-	-	2012-2014	PFS

[1]Only median is reported

[2]https://cancerres.aacrjournals.org/content/73/24_Supplement/S3-07.abstract

Regimen naming conventions: The character *and* (&) indicates that treatments are given together in combination; the character *slash* (/) indicates that treatments are alternated; the character *dash* (-) indicates that treatments are given sequentially. Numbers are used to delineate regimens with the same drugs but different doses; these numbers correspond to baseline dosing with the standard units of measurement for the given drug (e.g., docetaxel 75 = docetaxel 75 mg/m2; bevacizumab 15 = bevacizumab 15 mg/kg). The character "x" indicates that a regimen is repeated for a number of cycles (e.g., x 3 means that a regimen is repeated 3 times total); in the absence of this notation, it can generally be assumed that treatment is indefinite until progression of disease or excess toxicity; qwk: treatment is repeated weekly; q3wk: treatment is repeated every 3 weeks.

Regimen abbreviations: A: doxorubicin; AC: cyclophosphamide & doxorubicin; ACT: doxorubicin, cyclophosphamide, tamoxifen; AF: doxorubicin & fluorouracil; AFCM: doxorubicin, fluorouracil, cyclophosphamide, methotrexate; AG: aminoglutethemide; ALP: alpelisib; AT(Taxol): doxorubicin & paclitaxel; AT(Taxol): doxorubicin & paclitaxel; AT(Taxotere): docetaxel & doxorubicin; AV: doxorubicin & vincristine; A-CMFVP: doxorubicin followed by CMFVP; A-D: doxorubicin followed by docetaxel; A-D-PLD: doxorubicin followed by docetaxel followed by PLD; BCG: bacillus Calmette-Guérin; BMF: bendamustine, methotrexate, fluorouracil; CAF/FAC: cyclophosphamide, doxorubicin, fluorouracil; CAFT: cyclophosphamide, doxorubicin, fluorouracil, tamoxifen; CAFTH: cyclophosphamide, doxorubicin, fluorouracil, tamoxifen, fluoxymesterone; CAFVP: cyclophosphamide, doxorubicin, fluorouracil, vincristine, prednisone; CEF/FEC: cyclophosphamide, epirubicin, fluorouracil; CFP: cyclophosphamide, fluorouracil, prednisone; CI: continuous infusion; CME: cyclophosphamide, mitomycin, etoposide; CMF: cyclophosphamide, methotrexate, fluorouracil; CMFP: cyclophosphamide, methotrexate, fluorouracil, prednisone; CMFT: cyclophosphamide, methotrexate, fluorouracil, tamoxifen; CMFVP: cyclophosphamide, methotrexate, fluorouracil, vincristine, prednisone; CNF: cyclophosphamide, mitoxantrone, fluorouracil; D: docetaxel; DE: docetaxel & epirubicin; DES: diethylstilbestrol; E: epirubicin; EC: cyclophosphamide & epirubicin; EM: epirubicin & mitomycin; EP: epirubicin & paclitaxel; E-P: epirubicin followed by paclitaxel; HC: hydrocortisone; HD: high-dose; LAR: long-acting release; MC: cyclophosphamide & non-pegylated liposomal doxorubicin; MPA: medroxyprogesterone acetate; NPLD: non-pegylated liposomal doxorubicin; O+CAF: oophorectomy, cyclophosphamide, doxorubicin, fluorouracil; P: paclitaxel; PE: cisplatin & etoposide; PLD: pegylated liposomal doxorubicin; SMF: prednimustine, methotrexate, fluorouracil; TAD: tamoxifen, aminoglutethimide, danazol; TBX: taxane, bevacizumab, capecitabine; TEX: paclitaxel, epirubicin, capecitabine; TI FEC: time-intensive

© 2022 Li X et al. *JAMA Network Open.*

fluorouracil, epirubicin, cyclophosphamide; TI MMM: time-intensive mitoxantrone, methotrexate, mitomycin C; TX(Taxotere): docetaxel & capecitabine; VAC: vincristine, doxorubicin, cyclophosphamide; VATH: vinblastine, doxorubicin, thiotepa, halotestin.

Enrollment abbreviation: NR: not reported

Endpoint abbreviations: CBR: clinical benefit rate; CRR: complete response rate; DCR: disease control rate; ORR: overall response rate; OS: overall survival; PFS: progression-free survival; PFS6: progression-free survival at 6 months; PFS12: progression-free survival at 12 months; TTF: time to treatment failure; TTP: time to progression

© 2022 Li X et al. *JAMA Network Open.*

eTable 2. Full Ranked List of Regimens by IT-NMA

Rank	Regimen	Value	Aging coefficient	Modality
1	Letrozole plus palbociclib	17.84	0.91	Endocrine and targeted therapy
2	Paclitaxel plus bevacizumab	15.92	0.64	Cytotoxic chemotherapy and targeted therapy
3	Letrozole plus ribociclib	11.87	0.78	Endocrine and targeted therapy
4	Anastrozole plus ribociclib	10.88	0.88	Endocrine and targeted therapy
5	Fulvestrant plus ribociclib	9.31	1.00	Endocrine and targeted therapy
6	Abemaciclib plus anastrozole	9.30	0.78	Endocrine and targeted therapy
7	Capecitabine plus paclitaxel plus bevacizumab	6.13	0.69	Cytotoxic chemotherapy and targeted therapy
8	Capecitabine 2000 mg/m2 plus docetaxel 75 mg/m2 (TX 2000/75), 8 cycles	4.77	0.60	Cytotoxic chemotherapy
9	Paclitaxel 80 mg/m2, weekly	4.51	0.42	Cytotoxic chemotherapy
10	Capecitabine plus sorafenib	4.04	0.41	Cytotoxic chemotherapy and targeted therapy
11	Taxane plus anthracycline plus bevacizumab	3.82	0.36	Cytotoxic chemotherapy and targeted therapy
12	Anastrozole plus fulvestrant	3.81	0.65	Endocrine therapy
13	Docetaxel 60 to 75 mg/m2, every 3-4 weeks	3.66	0.69	Cytotoxic chemotherapy
14	Letrozole plus bevacizumab	3.33	0.69	Endocrine and targeted therapy
15	Paclitaxel 80 mg/m2, 3 weeks out of 4	3.20	1.00	Cytotoxic chemotherapy
16 (tie)	Docetaxel plus PLD	3.01	0.78	Cytotoxic chemotherapy
16 (tie)	Polymeric micellar paclitaxel (Genexol-PM)	3.01	0.69	Cytotoxic chemotherapy
18	Doxorubicin followed by docetaxel followed by PLD (A-D-PLD)	2.76	0.28	Cytotoxic chemotherapy
19	Gemcitabine plus paclitaxel, 6 cycles	2.27	0.47	Cytotoxic chemotherapy
20	Capecitabine plus vinorelbine plus bevacizumab	2.24	0.69	Cytotoxic chemotherapy and targeted therapy
21	Epirubicin 35 mg/m2	2.15	0.17	Cytotoxic chemotherapy
22	Docetaxel 100 mg/m2 plus bevacizumab 15 mg/kg, 9 cycles	2.00	0.32	Cytotoxic chemotherapy and targeted therapy
23	NPLD 60 mg/m2 plus cyclophosphamide 600 mg/m2 (MC 60/600)	1.99	0.30	Cytotoxic chemotherapy

© 2022 Li X et al. *JAMA Network Open.*

24	Docetaxel plus ramucirumab	1.87	0.78	Cytotoxic chemotherapy and targeted therapy
25	Lapatinib plus letrozole	1.71	0.28	Endocrine and targeted therapy
26	Sapitinib 20 mg twice/day plus anastrozole	1.55	0.69	Endocrine and targeted therapy
27	Docetaxel plus epirubicin (DE), 8 cycles	1.38	0.26	Cytotoxic chemotherapy
28	Doxorubicin 50 mg/m2 plus paclitaxel 150 mg/m2 (AT 50/150)	1.35	0.13	Cytotoxic chemotherapy
29	Gemcitabine plus paclitaxel	1.34	0.51	Cytotoxic chemotherapy
30	Doxorubicin 75 mg/m2	1.23	0.10	Cytotoxic chemotherapy
31	Doxorubicin plus cyclophosphamide alternating with docetaxel (AC/D)	1.16	0.28	Cytotoxic chemotherapy
32	Capecitabine 2000 mg/m2/day, limited duration	1.14	0.53	Cytotoxic chemotherapy
33	nab-Paclitaxel 260 mg/m2	1.08	0.17	Cytotoxic chemotherapy
34	BMF	0.91	0.17	Cytotoxic chemotherapy
35	Paclitaxel plus sorafenib	0.87	0.47	Cytotoxic chemotherapy and targeted therapy
36	Doxorubicin 50 mg/m2 plus docetaxel 75 mg/m2 (AT 50/75), 6 cycles	0.86	0.22	Cytotoxic chemotherapy
37	Time-intensive FEC	0.83	0.13	Cytotoxic chemotherapy
38	Docetaxel 100 mg/m2 plus bevacizumab 7.5 mg/kg	0.74	0.32	Cytotoxic chemotherapy and targeted therapy
39	Doxorubicin 50 mg/m2 plus paclitaxel 175 mg/m2 (AT 50/175)	0.71	0.22	Cytotoxic chemotherapy
40	LEM	0.70	0.09	Cytotoxic chemotherapy
41 (tie)	Doxorubicin 50 mg/m2 plus docetaxel 75 mg/m2 (AT 50/75), 8 cycles	0.69	0.16	Cytotoxic chemotherapy
41 (tie)	Doxorubicin 50 mg/m2 plus paclitaxel 220 mg/m2 (AT 50/220)	0.69	0.10	Cytotoxic chemotherapy
43 (tie)	Epirubicin plus vinorelbine	0.68	0.15	Cytotoxic chemotherapy
43 (tie)	TEX	0.68	0.36	Cytotoxic chemotherapy
45 (tie)	Letrozole plus temsirolimus	0.67	0.41	Endocrine and targeted therapy
45 (tie)	NPLD 75 mg/m2 plus cyclophosphamide 600 mg/m2 (MC 75/600)	0.67	0.17	Cytotoxic chemotherapy
47 (tie)	Docetaxel 80 mg/m2	0.65	0.36	Cytotoxic chemotherapy

© 2022 Li X et al. *JAMA Network Open.*

47 (tie)	Paclitaxel plus bevacizumab plus gemcitabine	0.65	0.69	Cytotoxic chemotherapy and targeted therapy
49	Fluorouracil 1000 mg/m2 plus epirubicin 100 mg/m2 plus cyclophosphamide 800 mg/m2 (FEC 1000/100/800)	0.60	0.10	Cytotoxic chemotherapy
50	Capecitabine 2000 mg/m2 plus docetaxel 75 mg/m2 (TX 2000/75), indefinite	0.59	0.46	Cytotoxic chemotherapy
51	Docetaxel plus sunitinib	0.58	0.41	Cytotoxic chemotherapy and targeted therapy
52	Cisplatin plus epirubicin plus lonidamine	0.57	0.12	Cytotoxic chemotherapy
53	Capecitabine 1300 mg/m2/day	0.55	0.36	Cytotoxic chemotherapy
54	Paclitaxel plus lapatinib	0.54	0.25	Cytotoxic chemotherapy and targeted therapy
55	Cisplatin plus epirubicin	0.51	0.12	Cytotoxic chemotherapy
56	Exemestane	0.50	0.33	Endocrine therapy
57	Sapitinib 40 mg twice/day plus anastrozole	0.48	0.69	Endocrine and targeted therapy
58	Goserelin plus tamoxifen	0.44	0.05	Endocrine therapy
59	Epirubicin 135 mg/m2	0.43	0.07	Cytotoxic chemotherapy
60	Taxane plus bevacizumab	0.41	0.60	Cytotoxic chemotherapy and targeted therapy
61 (tie)	Gefitinib plus tamoxifen	0.40	0.36	Endocrine and targeted therapy
61 (tie)	nab-Paclitaxel 100 mg/m2	0.40	0.38	Cytotoxic chemotherapy
63	Paclitaxel 250 mg/m2, every 3 weeks	0.31	0.21	Cytotoxic chemotherapy
64 (tie)	Fluorouracil 500 mg/m2 plus epirubicin 60 mg/m2 plus cyclophosphamide 500 mg/m2 (FEC 500/60/500)	0.30	0.04	Cytotoxic chemotherapy
64 (tie)	PLD 50 mg/m2	0.30	0.41	Cytotoxic chemotherapy
66	FEC followed by high-dose chemotherapy with stem cell transplant	0.29	0.24	Cytotoxic chemotherapy
67	nab-Paclitaxel 150 mg/m2	0.28	0.63	Cytotoxic chemotherapy
68 (tie)	LFEC	0.27	0.09	Cytotoxic chemotherapy
68 (tie)	Motesanib plus paclitaxel	0.27	0.49	Cytotoxic chemotherapy and targeted therapy

© 2022 Li X et al. *JAMA Network Open.*

70	Fluorouracil 500 mg/m2 plus epirubicin 50 mg/m2 plus cyclophosphamide 500 mg/m2 (FEC 500/50/500)	0.26	0.14	Cytotoxic chemotherapy
71 (tie)	CAFTH	0.24	0.09	Cytotoxic chemotherapy and endocrine therapy
71 (tie)	Fluorouracil 1000 mg/m2 plus epirubicin 60 mg/m2 plus oral cyclophosphamide 1400 mg/m2 (FEC 1000/60/1400)	0.24	0.08	Cytotoxic chemotherapy
73 (tie)	Doxorubicin plus MPA	0.21	0.04	Cytotoxic chemotherapy and endocrine therapy
73 (tie)	VATH/CMFVP	0.21	0.07	Cytotoxic chemotherapy and endocrine therapy
75	FEC for 18 months	0.18	0.04	Cytotoxic chemotherapy
76	Epirubicin followed by paclitaxel (E-P)	0.16	0.15	Cytotoxic chemotherapy
77	Toremifene 240 mg/day	0.14	0.15	Endocrine therapy
78 (tie)	Idoxifene	0.13	0.22	Endocrine therapy
78 (tie)	MPA 1000 mg/day	0.13	0.22	Endocrine therapy
80 (tie)	Gemcitabine plus epirubicin plus paclitaxel	0.12	0.17	Cytotoxic chemotherapy
80 (tie)	MPA	0.12	0.18	Endocrine therapy
82 (tie)	Epirubicin 90 mg/m2	0.11	0.13	Cytotoxic chemotherapy
82 (tie)	Pictilisib plus paclitaxel	0.11	0.69	Cytotoxic chemotherapy and targeted therapy
84	Tamoxifen alternating with MPA	0.10	0.03	Endocrine therapy
85 (tie)	CAFVP	0.09	0.02	Cytotoxic chemotherapy
85 (tie)	Capecitabine 1900 mg/m2 plus docetaxel 75 mg/m2 (TX 1900/75)	0.09	0.28	Cytotoxic chemotherapy
85 (tie)	CMFT	0.09	0.03	Cytotoxic chemotherapy and endocrine therapy
85 (tie)	Toremifene 60 mg/day	0.09	0.15	Endocrine therapy
89 (tie)	CAF plus MPA	0.08	0.04	Cytotoxic chemotherapy and endocrine therapy
89 (tie)	Docetaxel 100 mg/m2, 6 cycles	0.08	0.19	Cytotoxic chemotherapy
89 (tie)	Doxorubicin plus etoposide	0.08	0.08	Cytotoxic chemotherapy

© 2022 Li X et al. *JAMA Network Open.*

89 (tie)	Fluorouracil 1000 mg/m2 plus doxorubicin 50 mg/m2 plus oral cyclophosphamide 1400 mg/m2 (FAC 1000/50/1400)	0.08	0.02	Cytotoxic chemotherapy
93	Tamoxifen 20 mg/day plus ovarian irradiation plus prednisolone	0.07	0.02	Endocrine therapy
94	Celecoxib plus exemestane	0.06	0.25	Endocrine therapy
95	Paclitaxel plus bevacizumab plus everolimus	0.05	0.69	Cytotoxic chemotherapy and targeted therapy
96	TAD	0.04	0.22	Cytotoxic chemotherapy and endocrine therapy
97	CMFVP weekly	0.03	0.02	Cytotoxic chemotherapy and endocrine therapy
98 (tie)	Aminoglutethemide plus hydrocortisone plus tamoxifen	0.02	0.22	Endocrine therapy
98 (tie)	Atamestane plus toremifene	0.02	0.22	Endocrine therapy
98 (tie)	Bilateral oophorectomy	0.02	0.25	Endocrine therapy
98 (tie)	Cyclophosphamide 100 mg/m2 plus methotrexate 40 mg/m2 plus fluorouracil 600 mg/m2 (CMF 100/40/600)	0.02	0.01	Cytotoxic chemotherapy
98 (tie)	FAC-BCG	0.02	0.01	Cytotoxic chemotherapy and immunotherapy
98 (tie)	O+CAF	0.02	0.04	Cytotoxic chemotherapy and endocrine therapy
98 (tie)	Trebananib 10 mg/kg plus paclitaxel plus bevacizumab	0.02	0.58	Cytotoxic chemotherapy and targeted therapy
105 (tie)	CFP	0.01	0.02	Cytotoxic chemotherapy and endocrine therapy
105 (tie)	CMFVP	0.01	0.04	Cytotoxic chemotherapy and endocrine therapy
105 (tie)	DES	0.01	0.22	Endocrine therapy
105 (tie)	VAC	0.01	0.03	Cytotoxic chemotherapy
105 (tie)	VATH	0.01	0.07	Cytotoxic chemotherapy and endocrine therapy
110 (tie)	ACT	0.00	0.10	Cytotoxic chemotherapy and endocrine therapy
110 (tie)	AFCM	0.00	0.01	Cytotoxic chemotherapy

© 2022 Li X et al. *JAMA Network Open.*

110 (tie)	Aminoglutethimide	0.00	0.22	Endocrine therapy
110 (tie)	Anastrozole plus gefitinib	0.00	0.38	Endocrine and targeted therapy
110 (tie)	Buparlisib plus paclitaxel	0.00	0.78	Cytotoxic chemotherapy and targeted therapy
110 (tie)	CAFT	0.00	0.02	Cytotoxic chemotherapy and endocrine therapy
110 (tie)	Capecitabine 2500 mg/m2 plus docetaxel 75 mg/m2 (TX 2500/75)	0.00	0.36	Cytotoxic chemotherapy
110 (tie)	Capecitabine 2510 mg/m2/day	0.00	0.10	Cytotoxic chemotherapy
110 (tie)	Capecitabine plus paclitaxel	0.00	0.47	Cytotoxic chemotherapy
110 (tie)	Cisplatin plus etoposide	0.00	0.06	Cytotoxic chemotherapy
110 (tie)	Cyclophosphamide plus mitomycin plus etoposide	0.00	0.17	Endocrine and targeted therapy
110 (tie)	Dasatinib plus letrozole	0.00	0.47	Endocrine and targeted therapy
110 (tie)	DES-CEF	0.00	0.05	Cytotoxic chemotherapy and endocrine therapy
110 (tie)	Docetaxel 25 mg/m2	0.00	0.36	Cytotoxic chemotherapy
110 (tie)	Docetaxel 36 mg/m2	0.00	0.28	Cytotoxic chemotherapy
110 (tie)	Docetaxel 40 mg/m2	0.00	0.41	Cytotoxic chemotherapy
110 (tie)	Docetaxel 75 mg/m2 plus bevacizumab 15 mg/kg	0.00	0.69	Cytotoxic chemotherapy and targeted therapy
110 (tie)	Docetaxel plus bevacizumab, followed by exemestane plus bevacizumab	0.00	0.69	Cytotoxic chemotherapy and endocrine therapy and targeted therapy
110 (tie)	Doxorubicin 40 mg/m2 plus oral cyclophosphamide 800 mg/m2 (AC 40/800 PO)	0.00	0.01	Cytotoxic chemotherapy
110 (tie)	Doxorubicin 50 mg/m2 plus cyclophosphamide 750 mg/m2 (AC 50/750)	0.00	0.07	Cytotoxic chemotherapy
110 (tie)	Doxorubicin followed by CMFVP (A-CMFVP)	0.00	0.02	Cytotoxic chemotherapy
110 (tie)	Doxorubicin plus fluorouracil (AF)	0.00	0.01	Cytotoxic chemotherapy
110 (tie)	Epirubicin 60 mg/m2 plus paclitaxel 175 mg/m2 (EP 60/175)	0.00	0.47	Cytotoxic chemotherapy
110 (tie)	Fluorouracil 600 mg/m2 plus doxorubicin 50 mg/m2 plus cyclophosphamide 600 mg/m2 (FAC 600/50/600)	0.00	0.08	Cytotoxic chemotherapy

© 2022 Li X et al. *JAMA Network Open.*

110 (tie)	Fluorouracil 800 mg/m2 plus doxorubicin 40 mg/m2 plus cyclophosphamide 400 mg/m2 (FAC 800/40/400)	0.00	0.01	Cytotoxic chemotherapy
110 (tie)	Fluoxymesterone plus tamoxifen	0.00	0.36	Endocrine therapy
110 (tie)	Fulvestrant plus gefitinib	0.00	0.41	Endocrine and targeted therapy
110 (tie)	HD-CEF14	0.00	0.10	Cytotoxic chemotherapy
110 (tie)	Intermittent tamoxifen plus MPA	0.00	0.19	Endocrine therapy
110 (tie)	Ixabepilone every 3 weeks plus bevacizumab	0.00	0.57	Cytotoxic chemotherapy and targeted therapy
110 (tie)	Lomustine	0.00	0.02	Cytotoxic chemotherapy
110 (tie)	Melphalan	0.00	0.36	Cytotoxic chemotherapy
110 (tie)	Paclitaxel 210 mg/m2	0.00	0.15	Cytotoxic chemotherapy
110 (tie)	Paclitaxel plus sunitinib	0.00	0.60	Cytotoxic chemotherapy and targeted therapy
110 (tie)	PLD 40 mg/m2	0.00	0.28	Cytotoxic chemotherapy
110 (tie)	Semustine	0.00	0.02	Cytotoxic chemotherapy
110 (tie)	SMF	0.00	0.08	Cytotoxic chemotherapy and endocrine therapy
110 (tie)	Sunitinib plus paclitaxel plus bevacizumab	0.00	0.60	Cytotoxic chemotherapy and targeted therapy
110 (tie)	Time-intensive MMM	0.00	0.13	Cytotoxic chemotherapy
110 (tie)	Toremifene 200 mg/day	0.00	0.16	Endocrine therapy
110 (tie)	Vinorelbine 25 mg/m2 plus capecitabine 1650 mg/m2/day	0.00	0.36	Cytotoxic chemotherapy
151 (tie)	Doxorubicin 50 mg/m2 plus weekly docetaxel 75 mg/m2 (AT 50/75 qwk), 8 cycles	-0.01	0.32	Cytotoxic chemotherapy
151 (tie)	FAC plus leucovorin	-0.01	0.13	Cytotoxic chemotherapy
151 (tie)	Fluorouracil 1000 mg/m2 plus doxorubicin 60 mg/m2 plus oral cyclophosphamide 1400 mg/m2 (FAC 1000/60/1400), until cumulative doxorubicin of 500 mg/m2	-0.01	0.03	Cytotoxic chemotherapy
151 (tie)	Fluorouracil 1000 mg/m2 plus epirubicin 50 mg/m2 plus cyclophosphamide 500 mg/m2 (FEC 1000/50/500)	-0.01	0.08	Cytotoxic chemotherapy
151 (tie)	Nandrolone	-0.01	0.13	Endocrine therapy

© 2022 Li X et al. *JAMA Network Open.*

156 (tie)	CMF for 6 cycles, followed by intensification	-0.02	0.36	Cytotoxic chemotherapy
156 (tie)	Cyclophosphamide 600 mg/m2 plus methotrexate 40 mg/m2 plus fluorouracil 600 mg/m2 (CMF 600/40/600)	-0.02	0.19	Cytotoxic chemotherapy
156 (tie)	Epirubicin plus vindesine	-0.02	0.06	Cytotoxic chemotherapy
156 (tie)	Fluorouracil 1000 mg/m2 plus doxorubicin 50 mg/m2 plus cyclophosphamide 500 mg/m2 (FAC 1000/50/500)	-0.02	0.01	Cytotoxic chemotherapy
160	Megestrol plus tamoxifen	-0.03	0.09	Endocrine therapy
161 (tie)	Doxorubicin 60 mg/m2 plus paclitaxel 200 mg/m2 (AT 60/200)	-0.05	0.14	Cytotoxic chemotherapy
161 (tie)	Intermittent tamoxifen	-0.05	0.19	Endocrine therapy
161 (tie)	Mitoxantrone	-0.05	0.12	Cytotoxic chemotherapy
161 (tie)	Octreotide LAR plus tamoxifen	-0.05	0.36	Endocrine therapy
165 (tie)	Doxorubicin 40 mg/m2 plus cyclophosphamide 400 mg/m2 (AC 40/400)	-0.06	0.02	Cytotoxic chemotherapy
165 (tie)	Epirubicin 50 mg/m2	-0.06	0.04	Cytotoxic chemotherapy
165 (tie)	Fluorouracil 1000 mg/m2 plus doxorubicin 60 mg/m2 plus oral cyclophosphamide 1400 mg/m2 (FAC 1000/60/1400), indefinite	-0.06	0.05	Cytotoxic chemotherapy
165 (tie)	Tamoxifen plus prednisolone	-0.06	0.36	Endocrine therapy
169 (tie)	Doxorubicin plus vincristine (AV)	-0.07	0.17	Cytotoxic chemotherapy
169 (tie)	Fadrozole	-0.07	0.36	Endocrine therapy
169 (tie)	Tamoxifen 20 mg/day plus ovarian irradiation	-0.07	0.02	Endocrine therapy
172 (tie)	Doxorubicin plus vinorelbine	-0.09	0.12	Cytotoxic chemotherapy
172 (tie)	Epirubicin 70 mg/m2	-0.09	0.12	Cytotoxic chemotherapy
174	Tamoxifen 30 mg/day	-0.10	0.03	Endocrine therapy
175	CMF plus MPA	-0.11	0.19	Cytotoxic chemotherapy and endocrine therapy
176 (tie)	Epirubicin 90 mg/m2 plus cyclophosphamide 600 mg/m2 (EC 90/600)	-0.12	0.32	Cytotoxic chemotherapy
176 (tie)	Fluorouracil 500 mg/m2 plus epirubicin 90 mg/m2 plus cyclophosphamide 500 mg/m2 (FEC 500/90/500)	-0.12	0.17	Cytotoxic chemotherapy
176 (tie)	Low-dose CMF	-0.12	0.03	Cytotoxic chemotherapy

© 2022 Li X et al. *JAMA Network Open.*

179 (tie)	Epirubicin plus lonidamine	-0.15	0.12	Cytotoxic chemotherapy
179 (tie)	Megestrol	-0.15	0.25	Endocrine therapy
181 (tie)	CMF for 4 to 6 cycles, followed by high-dose therapy	-0.16	0.36	Cytotoxic chemotherapy
181 (tie)	Doxorubicin 20 mg/m2	-0.16	0.04	Cytotoxic chemotherapy
181 (tie)	Epirubicin 75 mg/m2 plus paclitaxel 200 mg/m2 (EP 75/200)	-0.16	0.17	Cytotoxic chemotherapy
181 (tie)	Epirubicin 90 mg/m2 plus paclitaxel 200 mg/m2 (EP 90/200)	-0.16	0.15	Cytotoxic chemotherapy
185	Oral cyclophosphamide 1400 mg/m2 plus methotrexate 80 mg/m2 plus fluorouracil 1000 mg/m2 (CMF 1400/80/1000)	-0.17	0.02	Cytotoxic chemotherapy
186	Epirubicin plus mitomycin (EM)	-0.18	0.09	Cytotoxic chemotherapy
187 (tie)	Fluorouracil 500 mg/m2 plus epirubicin 100 mg/m2 plus cyclophosphamide 500 mg/m2 (FEC 500/100/500)	-0.19	0.19	Cytotoxic chemotherapy
187 (tie)	Trebananib 3 mg/kg plus paclitaxel plus bevacizumab	-0.19	0.56	Cytotoxic chemotherapy and targeted therapy
189 (tie)	CMFP	-0.21	0.06	Cytotoxic chemotherapy and endocrine therapy
189 (tie)	Fluorouracil 1000 mg/m2 plus doxorubicin 40 mg/m2 plus cyclophosphamide 500 mg/m2 (FAC 1000/40/500)	-0.21	0.11	Cytotoxic chemotherapy
191	Fluorouracil 600 mg/m2 plus epirubicin 60 mg/m2 plus cyclophosphamide 600 mg/m2 (FEC 600/60/600)	-0.23	0.05	Cytotoxic chemotherapy
192 (tie)	CNF	-0.24	0.08	Cytotoxic chemotherapy
192 (tie)	Fluorouracil 1000 mg/m2 plus doxorubicin 60 mg/m2 plus oral cyclophosphamide 1400 mg/m2 (FAC 1000/60/1400), 6 cycles	-0.24	0.09	Cytotoxic chemotherapy
192 (tie)	Fluorouracil 600 mg/m2 plus epirubicin 70 mg/m2 plus cyclophosphamide 600 mg/m2 (FEC 600/70/600)	-0.24	0.25	Cytotoxic chemotherapy
195	Epirubicin 80 mg/m2 plus paclitaxel 175 mg/m2 (EP 80/175)	-0.26	0.25	Cytotoxic chemotherapy
196	Carboplatin plus paclitaxel	-0.29	0.32	Cytotoxic chemotherapy

© 2022 Li X et al. *JAMA Network Open.*

197 (tie)	Capecitabine 2500 mg/m2/day	-0.30	0.69	Cytotoxic chemotherapy
197 (tie)	FEC weekly	-0.30	0.04	Cytotoxic chemotherapy
199	Paclitaxel 250 mg/m2, over 3 hours	-0.31	0.08	Cytotoxic chemotherapy
200	NPLD	-0.38	0.12	Cytotoxic chemotherapy
201	Docetaxel plus epirubicin (DE)	-0.40	0.29	Cytotoxic chemotherapy
202	Taxane plus bevacizumab plus capecitabine	-0.41	0.60	Cytotoxic chemotherapy and targeted therapy
203 (tie)	Fulvestrant 250 mg	-0.42	0.36	Endocrine therapy
203 (tie)	nab-Paclitaxel 300 mg/m2	-0.42	0.38	Cytotoxic chemotherapy
205	Epirubicin 75 mg/m2	-0.43	0.10	Cytotoxic chemotherapy
206 (tie)	Epirubicin 40 mg/m2	-0.44	0.07	Cytotoxic chemotherapy
206 (tie)	Goserelin	-0.44	0.05	Endocrine therapy
208	Epirubicin 75 mg/m2 plus cyclophosphamide 600 mg/m2 (EC 75/600)	-0.51	0.17	Cytotoxic chemotherapy
209	Doxorubicin 60 mg/m2	-0.55	0.14	Cytotoxic chemotherapy
210 (tie)	Doxorubicin 40 mg/m2 plus cyclophosphamide 500 mg/m2 (AC 40/500)	-0.57	0.28	Cytotoxic chemotherapy
210 (tie)	Paclitaxel 200 mg/m2	-0.57	0.09	Cytotoxic chemotherapy
210 (tie)	Trebananib 10 mg/kg plus paclitaxel	-0.57	0.56	Cytotoxic chemotherapy and targeted therapy
213	Docetaxel 60 mg/m2, every 3 weeks	-0.59	0.28	Cytotoxic chemotherapy
214	Doxorubicin 60 mg/m2 plus cyclophosphamide 600 mg/m2 (AC 60/600)	-0.64	0.13	Cytotoxic chemotherapy
215	Axitinib plus docetaxel	-0.65	0.36	Cytotoxic chemotherapy and targeted therapy
216	Epirubicin 75 mg/m2 plus paclitaxel 175 mg/m2 (EP 75/175)	-0.68	0.36	Cytotoxic chemotherapy
217	Doxorubicin 50 mg/m2 plus docetaxel 75 mg/m2 (AT 50/75), 4 cycles	-0.71	0.22	Cytotoxic chemotherapy
218	Epirubicin 60 mg/m2	-0.76	0.07	Cytotoxic chemotherapy
219	Paclitaxel 175 mg/m2, continuous infusion	-0.80	0.13	Cytotoxic chemotherapy
220	Tamoxifen 20 mg/day	-0.83	0.38	Endocrine therapy
221	Epirubicin 120 mg/m2	-0.84	0.12	Cytotoxic chemotherapy
222	Docetaxel plus gemcitabine	-1.02	0.46	Cytotoxic chemotherapy

© 2022 Li X et al. *JAMA Network Open.*

223	Sepantronium plus docetaxel	-1.12	0.60	Cytotoxic chemotherapy and targeted therapy
224 (tie)	Droloxifene	-1.14	0.36	Endocrine therapy
224 (tie)	PLD 45 mg/m2	-1.14	0.53	Cytotoxic chemotherapy
226	Docetaxel 75 mg/m2	-1.18	0.74	Cytotoxic chemotherapy
227	Capecitabine plus bevacizumab	-1.26	0.66	Cytotoxic chemotherapy and targeted therapy
228	Capecitabine plus bevacizumab plus cyclophosphamide	-1.27	0.69	Cytotoxic chemotherapy and targeted therapy
229	Arzoxifene	-1.41	0.36	Endocrine therapy
230	Tamoxifen 40 mg/day	-1.48	0.23	Endocrine therapy
231	Fluorouracil 500 mg/m2 plus doxorubicin 50 mg/m2 plus cyclophosphamide 500 mg/m2 (FAC 500/50/500)	-1.62	0.08	Cytotoxic chemotherapy
232	Docetaxel 30 mg/m2	-1.80	0.60	Cytotoxic chemotherapy
233	NPLD plus vinorelbine	-1.98	0.53	Cytotoxic chemotherapy
234	nab-Paclitaxel plus bevacizumab	-2.01	0.60	Cytotoxic chemotherapy and targeted therapy
235	Gemcitabine	-2.15	0.17	Cytotoxic chemotherapy
236	Observation	-2.27	0.47	Null therapy
237	Oral cyclophosphamide 1400 mg/m2 plus methotrexate 80 mg/m2 plus fluorouracil 1200 mg/m2 (CMF 1400/80/1200)	-2.30	0.24	Cytotoxic chemotherapy
238	Fluorouracil 500 mg/m2 plus epirubicin 75 mg/m2 plus cyclophosphamide 500 mg/m2 (FEC 500/75/500)	-2.51	0.13	Cytotoxic chemotherapy
239	Doxorubicin followed by docetaxel (A-D)	-2.63	0.28	Cytotoxic chemotherapy
240	Docetaxel 100 mg/m2, 9 cycles	-2.74	0.32	Cytotoxic chemotherapy
241	Docetaxel 100 mg/m2	-2.88	0.39	Cytotoxic chemotherapy
242	NK105 (micellar paclitaxel)	-3.20	1.00	Cytotoxic chemotherapy
243	S-1 (tegafur/gimeracil/oteracil)	-3.66	0.69	Cytotoxic chemotherapy
244	Taxane plus anthracycline	-3.82	0.36	Cytotoxic chemotherapy
245	Fulvestrant 500 mg	-4.11	0.94	Endocrine therapy
246	Vinorelbine plus capecitabine	-4.77	0.64	Cytotoxic chemotherapy

© 2022 Li X et al. *JAMA Network Open.*

247	Ixabepilone weekly plus bevacizumab	-6.24	0.59	Cytotoxic chemotherapy and targeted therapy
248	Capecitabine 2000 mg/m2/day	-7.03	0.57	Cytotoxic chemotherapy
249	Paclitaxel 175 mg/m2, every 3 weeks	-8.01	0.32	Cytotoxic chemotherapy
250	Paclitaxel 90 mg/m2	-11.17	0.62	Cytotoxic chemotherapy
251	Anastrozole	-29.36	0.70	Endocrine therapy
252	Letrozole	-33.27	0.54	Endocrine therapy

[1]Tied ranks are denoted as such.

[2]See above (p.13) for regimen abbreviations.

eAppendix. Videos

Video 1. The Changing Value Rankings of the Regimen Network From 1974 to 2019

As the field expanded over time, more and more regimens were added to the regimen network. Clusters started to emerge among regimens being frequently compared to each other. This video depicts the value ranking of regimens over time, with the transparency of the nodes reflecting the salience of that regimen. https://youtu.be/Xz3JLVN

Video 2. The Changing Clustering of Regimen Modalities From 1974 to 2019

This video colors the nodes (regimens) by treatment modality, and demonstrates the emergence of clusters of more direct comparisons, over time. These clusters often consist of regimens of the same modality (e.g., a chemotherapy cluster and an endocrine therapy cluster). https://youtu.be/u1E64ttOOOE

© 2022 Li X et al. *JAMA Network Open.*
